# A repetitive amplitude encoding method for enhancing the mapping ability of quantum neural networks

**DOI:** 10.1038/s41598-025-17651-5

**Published:** 2025-09-01

**Authors:** Ziyang Li, Xiaofei Fu, Lingdong Meng, Ruishan Du

**Affiliations:** School of Earth Sciences, Northeast Petroleum University, Daqing, 163318 Heilongjiang China

**Keywords:** Computer science, Information technology

## Abstract

With the rapid development of quantum machine learning, quantum neural networks (QNNs) have become a research hotspot. However, the quantum gates used to implement feature mapping in this model are all linear transformations, which directly affects the mapping ability of the model. Therefore, how to enhance the mapping capability of QNN is an important issue that has not yet been effectively addressed. This paper proposes a repetitive amplitude encoding method that encodes the probability amplitudes of multiple qubit blocks by repeatedly using the same set of classical data, effectively improving the mapping capability of QNN. Taking the MNIST dataset as an example, the experimental results comparing the repetitive amplitude encoding method with several existing encoding methods show that, firstly, when the number of classes is fixed, the repetitive amplitude encoding is superior to other methods. Secondly, when the number of hidden layers in QNN is fixed, as the number of classes increases, the performance of repetitive amplitude encoding not only consistently outperforms other methods, but this advantage becomes increasingly apparent. Finally, the repetitive amplitude encoding-based QNN was applied to reservoir lithology identification in the field of oil and gas exploration, IRIS and WINe classification datasets. By comparing with classical neural networks, the proposed method was validated for its adaptability to different classification problems and superior classification performance compared to classical neural networks.

## Introduction

In recent years, quantum computing has achieved significant progress, deeply influencing various fields, including machine learning. Quantum computing operates on quantum bits (qubits), which can exist in superpositions of states, enabling parallel computation^1^.

The quantum neural network integrates quantum layers within a feed forward network structure^2,3^. These quantum layers perform operations like quantum gate transformations, enhancing the model’s expressiveness and learning capabilities. For example, quantum convolutional neural networks apply quantum analogs of convolutional operations to process data^4–6^. Training quantum neural network involves optimizing both quantum and classical parameters. Variational quantum algorithms (VQAs), such as the variational quantum eigensolver (VQE), and quantum approximate optimization algorithm (QAOA), are commonly used for this purpose^7,8^. These algorithms iteratively adjust the parameters of quantum circuits to minimize a cost function. Gradient-based optimization methods, like the quantum natural gradient descent (QNGD), have also been adapted for training quantum neural network^9^. These methods leverage the unique properties of quantum systems to efficiently navigate the parameter space and achieve optimal solutions.

Quantum neural network has shown promise in various classification tasks. In image classification, these networks have demonstrated superior performance in recognizing patterns and features compared to purely classical models^10^. For example, QNN models has been applied to the MNIST dataset, achieving high accuracy in digits recognition^10^. In addition to image classification, quantum neural network has been explored in natural language processing (NLP). Tasks such as text classification and sentiment analysis have benefited from the enhanced processing capabilities of quantum models^11,12^. In addition, quantum neural networks have also been successfully applied in audio steganography^13–15^.

Despite their potential, quantum neural network faces several challenges. A major issue is that the feature mapping of quantum neural networks relies on quantum gates that can only achieve linear transformations, which seriously weakens the ability of nonlinear mapping. Additionally, another significant challenge that QNN faces is the barren plateau phenomenon exhibited by the loss function during the training process of QNN, which becomes particularly evident as the depth of quantum circuits increases^16–20^. In terms of hardware implementation, a prominent issue is the noise and decoherence in quantum systems, which can degrade the performance of quantum circuits^21^. Strategies such as error correction and noise mitigation are critical for maintaining the fidelity of quantum computations^22^.

In quantum neural networks, the mapping relationship between input and output is mainly determined by parameterized quantum circuits composed of various quantum gates. In quantum computing, various quantum gates are unitary operators, which can be mathematically described by a unitary matrix. A parameterized quantum circuit composed of many quantum gates is essentially equivalent to a more complex unitary operator, which can also be described by a unitary matrix. Therefore, the input-output relationship of quantum neural networks is mainly described by unitary matrices. And any transformation performed through a matrix is a linear transformation. Although the measurement operation at the output end has nonlinearity, the measurement result that generates collapse based on probability has a high degree of randomness. Therefore, the nonlinear mapping ability of quantum neural networks is relatively weak. We believe that the problem of barren plateau in QNN training is related to the weak mapping ability of the network.

The encoding method from classical data to quantum data brings hope for introducing nonlinearity in quantum neural networks. The most commonly used encoding methods currently include amplitude encoding^23–28^, angle encoding^29–33^, and hybrid encoding^34^. Preprocessing steps, such as normalization and dimensionality reduction, are often necessary to prepare data for encoding. Techniques like principal component analysis (PCA), singular value decomposition (SVD) can be employed to reduce the complexity of data and improve the efficiency of quantum encoding^35,36^.

Amplitude encoding directly maps classical data to the probability amplitudes of the basis state in quantum systems, without introducing any nonlinearity, but it requires the minimum number of qubits. For an *n*-dimensional vector containing *n* data points, amplitude encoding requires $\lceil \log _{2}(n)\rceil$ qubits, which are implemented using uniformly controlled rotation. Employing uniformly controlled rotations, up to $2^{n+2}-4n-4$ CNOT gates and $2^{n+2}-5$ one-qubit elementary rotations are required to achieve perfect amplitude encoding. The complexity of the circuit is noticeably lower than the previously published results^37^.

The principle of angle encoding is to treat each classical data as the phase of a single qubit, and it cannot use the superposition of qubits to improve the loading capacity of classical data. Therefore, for *n* classical data, angle encoding requires *n* qubits^38^. Since angle encoding maps classical data to trigonometric values, the basis state probability amplitude of the encoded quantum system is the product of many trigonometric values, so this method can introduce a certain degree of nonlinear mapping capability. However, for multi qubit systems, the abnormally complex frequency characteristics presented by the product of multiple trigonometric functions disrupt the feature expression in the original data, thereby also affecting the improvement of QNN’s nonlinear mapping ability.

For hybrid encoding, qubits are first divided into *m* blocks, and then amplitude encoding is applied to each block of qubits. Therefore, the required number of qubits is $m\lceil \log _{2}(n/m)\rceil$, which falls between amplitude encoding and angle encoding. Although this method is also essentially amplitude encoding, by utilizing the superposition and entanglement effects between different qubit blocks, the probability amplitude of the entire quantum system after encoding can be the tensor product of each group of classical data. Not only did it achieve dimensional expansion, but it also brought about good nonlinear mapping. However, one drawback of this method is that when encoding each qubit block, the normalization constants of each group of classical data are inconsistent, resulting in the amplitudes may not be a faithful representation of the classical data unless the normalization constant have similar values.

For more complex problems, higher-order encoding techniques such as Z feature maps, ZZ feature maps, Pauli feature maps are also used^39^. However, these methods often target specific tasks and lack universality. Other encoding methods are often simple variations of existing methods. For example, kernel encoding^30^ is essentially angle encoding, just with a different name. Angle rotation encoding^40^ first transforms the numerical value *x* into $\theta =\arctan (x)$ or $\theta =\arctan (x^2)$, and then uses $\theta$ as the phase of qubit. The only difference from ordinary angle encoding is the way *x* is mapped to the interval $[0, \pi ]$. At present, the majority of literature uses angle encoding, followed by amplitude encoding, while the use of hybrid encoding is relatively rare.

In QNN, due to the fact that all quantum gates are unitary operators that can only perform linear transformations, the nonlinear mapping ability of the model is relatively weak. How to improve the nonlinear mapping ability of QNN is currently a key issue that has not been well addressed. This paper attempts to explore methods to improve the mapping capability of QNN from the perspective of classical data to quantum state encoding. Specifically, the encoding method proposed in this paper is an improvement on existing hybrid encoding methods, which can solve the drawback that hybrid encoding is not a faithful representation of classical data. Contributions stemming from this paper include:

- A repetitive amplitude encoding (RAE) method is designed, which can result in a non-linear transformation of classical data after encoding, thereby improving the mapping ability of QNN.
- A method for constructing a quantum neural network model based solely on quantum rotation gates and controlled NOT gates is provided.
- From binary classification to ten classification, a comprehensive examination is conducted to compare the classification performance of repetitive amplitude encoding with other encoding methods. The results show that as the number of classes gradually increases, the repetitive amplitude encoding shows a very obvious advantage.
- By applying the quantum neural network designed based on the proposed encoding method to reservoir lithology identification, the repetitive amplitude encoding method is promoted to engineering application.
- By applying the quantum neural network designed based on the proposed encoding method to the IRIS and WINE datasets, it is verified that the method is not only suitable for image recognition, but also for general classification and recognition problems.

## Method

### Qubit and quantum gates

In quantum computing, a qubit is a two-level quantum system, described by a vector in two-dimensional complex Hilbert space. From the superposition principles, any state of the qubit may be written as

1 $$\begin{aligned} |\varphi \rangle =\cos \frac{\theta }{2}|0\rangle +e^{\textrm{i}\phi }\sin \frac{\theta }{2}|1\rangle , \end{aligned}$$

where $$0\leqslant \theta \leqslant \uppi$$, $$0\leqslant \phi \leqslant 2\uppi$$.

Owing to the normalization condition, the qubit’s state can be represented by a point on a sphere of unit radius, called the Bloch Sphere. For convenience, in this paper, we represent the qubit’s state by a point on a circle of unit radius. At this time, any state of the qubit may be written as

2 $$\begin{aligned} |\varphi \rangle =\cos \alpha |0\rangle +\sin \alpha |1\rangle , \end{aligned}$$

The corresponding relations between Eqs. (1) and  (2) can be written as

3 $$\begin{aligned} \left\{ \begin{array}{cccccccccccccc} \alpha :& 0& \longrightarrow & \uppi /2& \Longleftrightarrow & \phi & =& 0& and& \theta :& \uppi /2& \longrightarrow & 0\\ \alpha :& \uppi /2& \longrightarrow & \uppi & \Longleftrightarrow & \phi & =& \uppi & and& \theta :& 0& \longrightarrow & \uppi /2\\ \alpha :& \uppi & \longrightarrow & 3\uppi /2& \Longleftrightarrow & \phi & =& \uppi & and& \theta :& \uppi /2& \longrightarrow & \uppi \\ \alpha :& 3\uppi /2& \longrightarrow & 2\uppi & \Longleftrightarrow & \phi & =& 0& and& \theta :& \uppi & \longrightarrow & \uppi /2 \end{array}\right. . \end{aligned}$$

In this paper, we employ the qubit representation given by Eq. (2) rather than that shown in Eq. (1) to construct the quantum neural network. Our rationale for this choice is explained as follows.

The use of qubits described by the unit circle in quantum neural networks is the result of the combined effects of hardware limitations in the NISQ era, model complexity control, and adaptation to task requirements. Although this simplification compresses the state space of qubits, it achieves a balance between effectiveness (task performance) and feasibility (experimental implementation) by retaining the core characteristic of phase interference, thus becoming the mainstream choice in current QNNs research and applications. With the development of quantum hardware (such as fault-tolerant quantum computers), it may gradually expand to the full Bloch sphere description in the future. However, at this stage, the unit circle simplification is the optimal solution that balances practicality and performance.

The *quantum gates* are the analogue of a logic gates in a classical computer, and they are basic units of quantum algorithms. The single qubit quantum gate used in this paper includes: Rotation gate *R*, Hadamard gate *H*, NOT gate *X*, Pauli *Z* gate, Identity gate *I*, and their definitions are as follows.

4 $$\begin{aligned} \left\{ \begin{array}{l} R(\alpha )= \begin{bmatrix} \cos \left( \frac{\alpha }{2}\right) & -\sin \left( \frac{\alpha }{2}\right) \\ \sin \left( \frac{\alpha }{2}\right) & ~~\cos \left( \frac{\alpha }{2}\right) \end{bmatrix}, H=\frac{1}{\sqrt{2}}\begin{bmatrix}1 & ~~1\\ 1 & -1\end{bmatrix},\\ \\ X=\begin{bmatrix} 0 & 1\\ 1 & 0\end{bmatrix}, ~~~~~Z=\begin{bmatrix}1 & ~~0\\ 0 & -1\end{bmatrix}, ~~~~~I=\begin{bmatrix}1 & 0\\ 0 & 1\end{bmatrix}. \end{array} \right. \end{aligned}$$

**Fig. 1:**
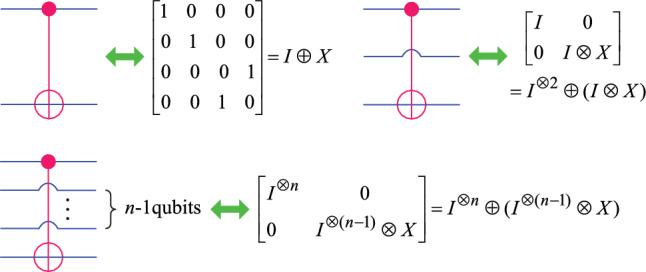
Symbol and matrix representation of controlled-NOT gates.

The multi-qubit gate used in this paper is a controlled-NOT gate (*CNOT*), and its quantum circuit and matrix representation are shown in Fig. 1.

### Direct sum and direct product of unitary operators

In quantum computing, any quantum gate is a unitary operator, which can be described using a unitary matrix. In the analysis of quantum circuits, the direct sum and direct product of unitary operators are two fundamental operations, represented by symbols $\oplus$ and $\otimes$, respectively.

Let $U_1$ and $U_2$ represent the matrix representation of two unitary operators, then their direct sum adds them as independent blocks along the diagonal, such as

5 $$\begin{aligned} U_1\oplus U_2=\begin{bmatrix}U_1 & 0\\ 0 & U_2\end{bmatrix}. \end{aligned}$$

Direct product is also called tensor product. It is a way of putting matrix spaces together to form larger matrix space. A direct product multiplies each element of the $U_1$ element-wise by the $U_2$, Such as

6 $$\begin{aligned} \begin{array}{ll} U_1=\begin{bmatrix}u_{1,1}^1 & \cdots & u_{1,m}^1\\ \vdots & \cdots & \vdots \\ u_{m,1}^1 & \cdots & u_{m,m}^1\end{bmatrix}, U_1\otimes U_2=\begin{bmatrix}u_{1,1}^1U_2 & \cdots & u_{1,m}^1U_2\\ \vdots & \cdots & \vdots \\ u_{m,1}^1U_2 & \cdots & u_{m,m}^1U_2\end{bmatrix}. \end{array} \end{aligned}$$

Direct sum and direct product are two important operators frequently used in the following part of this paper.

### Hybrid encoding

To illustrate the advantages of repetitive amplitude encoding, we first briefly describe the basic principles of hybrid encoding. Let *m* denote the number of qubits in each independent block that amplitude-encodes classical data. Then, each block can encode $2^m$ classical data. Let *b* denote that there are *b* such blocks of *m* qubits. Then, the quantum system of *b* blocks contains $b2^m$ classical data. Hybrid encoding can be expressed as^34^

7 $$\begin{aligned} U_\phi (x): x\in \mathbb {R}^N\rightarrow |\phi (x)\rangle =\bigotimes \limits _{j=1}^{b}\left( \frac{1}{\parallel x\parallel _j}\sum \limits _{i=1}^{2^m}x_{ij}|i-1\rangle _j\right) . \end{aligned}$$

Note that each block can have a different normalization constant, and hence, the amplitudes may not be a faithful representation of the data unless the normalization constant have similar values.

### Repetitive amplitude encoding

Please note that in hybrid encoding, due to the different normalization constants of each block, this will inevitably affect the matching degree between amplitude and sample data, resulting in amplitude not fully reflecting sample features. In this paper, repetitive amplitude encoding is proposed to address this issue. In this encoding method, different qubit blocks are encoded using the same set of classical data. Thus eliminating the problem of inconsistent normalization constants between different qubit blocks.

#### Principle of repetitive amplitude encoding

Let’s use *m* to represent the number of qubits used for amplitude encoding of classical data in each independent qubit block. Then, each block can encode $O(2^m)$ classical data. Let’s also denote that there are *n* such blocks of *m* qubits by *n*. Note that, at this time, these *n* blocks of *m* qubits are all encoded with the same classical data. Hence, the quantum system of *n* blocks only contains $2^m$ classical data. It is worth pointing out that although repetitive amplitude encoding reduces the data capacity of the quantum system, it can perfectly overcome the defects of inconsistent normalization constant of each qubit block inherent in general hybrid encoding.

Let $x=(x_1, x_2, \cdots , x_{2^m})^\text {T}$, the repetitive amplitude encoding can be expressed as follows.

8 $$\begin{aligned} U_\phi (x): x\in \mathbb {R}^N\rightarrow |\phi (x)\rangle =\bigotimes \limits _{j=1}^{n}\left( \frac{1}{\parallel x\parallel }\sum \limits _{i=1}^{2^m}x_{i}|i-1\rangle _j\right) . \end{aligned}$$

From the above equation, it can be seen that the normalization constants used in the *n* blocks of *m* qubits are exactly the same, which perfectly overcome the problem of inconsistent normalization constants in each qubit block.

To make it easier for readers to understand, we give an example below to specifically show the difference between repetitive amplitude encoding and hybrid encoding. For the classic data $\hat{x}^{(1)}=[\hat{x}_1, \hat{x}_2, \hat{x}_3, \hat{x}_4]^\text {T}$ and $\hat{x}^{(2)}=[\hat{x}_5, \hat{x}_6, \hat{x}_7, \hat{x}_8]^\text {T}$, let $x^{(1)}=[x_1, x_2, x_3, x_4]^\text {T}=\frac{1}{\parallel \hat{x}^{(1)}\parallel }(\hat{x}_1, \hat{x}_2, \hat{x}_3, \hat{x}_4)^\text {T}$, and $x^{(2)}=[x_5, x_6, x_7, x_8]^\text {T}=\frac{1}{\parallel \hat{x}^{(2)}\parallel }(\hat{x}_5, \hat{x}_6, \hat{x}_7, \hat{x}_8)^\text {T}$. Encoding $x^{(1)}$ and $x^{(2)}$ using hybrid encoding requires two 2-qubit blocks. The encoding result is as follows.

9 $$\begin{aligned} \begin{array}{lll} |\phi (x)\rangle & =& (x_1|00\rangle +x_2|01\rangle +x_3|10\rangle +x_4|11\rangle )\bigotimes (x_5|00\rangle +x_6|01\rangle +x_7|10\rangle +x_8|11\rangle ) \\ & =& \left( \begin{array}{l}x_1x_5|0000\rangle +x_1x_6|0001\rangle +x_1x_7|0010\rangle +x_1x_8|0011\rangle +\\ x_2x_5|0100\rangle +x_2x_6|0101\rangle +x_2x_7|0110\rangle +x_2x_8|0111\rangle +\\ x_3x_5|1000\rangle +x_3x_6|1001\rangle +x_3x_7|1010\rangle +x_3x_8|1011\rangle + \\ x_4x_5|1100\rangle +x_4x_6|1101\rangle +x_4x_7|1110\rangle +x_4x_8|1111\rangle \end{array}\right) . \end{array} \end{aligned}$$

Using repetitive amplitude encoding, two 2-qubit blocks are also required. Note that only 4 classic data can be encoded at this time. The encoding result is as follows.

10 $$\begin{aligned} \begin{array}{lll} |\phi (x)\rangle & =& (x_1|00\rangle +x_2|01\rangle +x_3|10\rangle +x_4|11\rangle )\bigotimes (x_1|00\rangle +x_2|01\rangle +x_3|10\rangle +x_4|11\rangle ) \\ & =& \left( \begin{array}{l}x_1^2|0000\rangle +x_1x_2|0001\rangle +x_1x_3|0010\rangle +x_1x_4|0011\rangle +\\ x_2x_1|0100\rangle +x_2^2|0101\rangle +x_2x_3|0110\rangle +x_2x_4|0111\rangle + \\ x_3x_1|1000\rangle +x_3x_2|1001\rangle +x_3^2|1010\rangle +x_3x_4|1011\rangle + \\ x_4x_1|1100\rangle +x_4x_2|1101\rangle +x_4x_3|1110\rangle +x_4^2|1111\rangle \end{array}\right) . \end{array} \end{aligned}$$

#### Advantages of repetitive amplitude encoding

- The repetitive amplitude encoding is the most faithful representation of the classical sample data

As to why the inconsistent normalization constants in each qubit block affect the fidelity of the amplitude to the sample data, we give an explanation from the perspective of the vector norm. Hybrid encoding is equivalent to dividing the original vector into multiple groups and then normalizing each group separately. Repetitive amplitude encoding is equivalent to repeatedly expanding the original vector into multiple groups and then normalizing each group separately. By calculating and comparing the norm of the difference between the vectors before and after grouping, it can be found that repetitive amplitude encoding has the best fidelity to the original data after grouping. This conclusion can be expressed as the following theorem.

##### Theorem

*Let* $X=[x_1,\cdots ,x_n,x_{n+1},\cdots ,x_{2n},\cdots ,x_{(m-1)n+1},\dots ,x_{mn}]$ *be an* *mn*-*dimensional vector, where* $n=2^t$. $X_1=\frac{[x_1,x_2,\cdots ,x_{mn}]}{\sqrt{x_1^2+x_2^2\cdots +x_{mn}^2}}$ *be the normalized vector of* *X*. *Divide* *X* *into* *m* *groups and let* $X_2=\left[ \frac{[x_1,\cdots ,x_n]}{\sqrt{x_1^2+\cdots +x_n^2}},\cdots ,\frac{[x_{(m-1)n+1},\cdots ,x_{mn}]}{\sqrt{x_{(m-1)n+1}^2+\cdots +x_{mn}^2}}\right]$ *be the grouped normalized vector that can encode* *m* *groups of qubits* $|q_{1,1}\cdots q_{1,t}\rangle \otimes \cdots \otimes |q_{m,1}\cdots q_{m,t}\rangle$ (*t* *in each group*). *Then the probability amplitude* $X_2$ *of* $|q_{1,1}\cdots q_{1,t}\rangle \otimes \cdots \otimes |q_{m,1}\cdots q_{m,t}\rangle$ *is the most faithful representation of the normalized vector* $X_1$ *when each group of qubits has the same normalization constant (i.e*. $x_1^2+\cdots +x_n^2=x_{n+1}^2+\cdots +x_{2n}^2=\cdots =x_{(m-1)n+1}^2+\cdots +x_{mn}^2$).

##### Proof

$$\begin{aligned} \begin{array}{lll} \Vert X_2-X_1\Vert & =& \left\| \left[ \frac{[x_1,\cdots ,x_n]}{\sqrt{x_1^2+\cdots +x_n^2}},\cdots ,\frac{[x_{(m-1)n+1},\cdots ,x_{mn}]}{\sqrt{x_{(m-1)n+1}^2+\cdots +x_{mn}^2}}\right] -\left[ \frac{[x_1,\cdots ,x_n]}{\sqrt{x_1^2+\cdots +x_{mn}^2}},\cdots ,\frac{[x_{(m-1)n+1},\cdots ,x_{mn}]}{\sqrt{x_1^2+\cdots +x_{mn}^2}}\right] \right\| \\ & =& \left\| \left[ \frac{[x_1,\cdots ,x_n]}{\sqrt{x_1^2+\cdots +x_n^2}}-\frac{[x_1,\cdots ,x_n]}{\sqrt{x_1^2+\cdots +x_{mn}^2}},\cdots ,\frac{[x_{(m-1)n+1},\cdots ,x_{mn}]}{\sqrt{x_{(m-1)n+1}^2+\cdots +x_{mn}^2}}-\frac{[x_{(m-1)n+1},\cdots ,x_{mn}]}{\sqrt{x_1^2+\cdots +x_{mn}^2}}\right] \right\| \\ & \ge & \left\| \left[ \frac{[x_1,\cdots ,x_n]}{\sqrt{x_1^2+\cdots +x_n^2}}-\frac{[x_1,\cdots ,x_n]}{\sqrt{m\root m \of {\left( \sum \limits _{i=1}^{n}x_i^2\right) \cdots \left( \sum \limits _{i=(m-1)n+1}^{mn}x_i^2\right) }}},\cdots ,\frac{[x_{(m-1)n+1},\cdots ,x_{mn}]}{\sqrt{x_{(m-1)n+1}^2+\cdots +x_{mn}^2}} \right. \right. \\ & & \left. \left. -\frac{[x_{(m-1)n+1},\cdots ,x_{mn}]}{\sqrt{m\root m \of {\left( \sum \limits _{i=1}^{n}x_i^2\right) \cdots \left( \sum \limits _{i=(m-1)n+1}^{mn}x_i^2\right) }}}\right] \right\| \\ \end{array} \end{aligned}$$

If and only if $x_1^2+\cdots +x_n^2=x_{n+1}^2+\cdots +x_{2n}^2=\cdots =x_{(m-1)n+1}^2+\cdots +x_{mn}^2$, the equal sign in the above equation holds. At this point, we can obtain

$$\begin{array}{lll} \Vert X_2-X_1\Vert _{min}& =& \left\| \left[ \frac{[x_1,\cdots ,x_n]}{\sqrt{x_1^2+\cdots +x_n^2}}-\frac{[x_1,\cdots ,x_n]}{\sqrt{m}\sqrt{x_1^2+\cdots +x_n^2}},\cdots ,\frac{[x_{(m-1)n+1},\cdots ,x_{mn}]}{\sqrt{x_1^2+\cdots +x_n^2}}-\frac{[x_{(m-1)n+1},\cdots ,x_{mn}]}{\sqrt{m}\sqrt{x_1^2+\cdots +x_n^2}}\right] \right\| \\ & =& \left( 1-\frac{1}{\sqrt{m}}\right) \left\| \left[ \frac{[x_1,\cdots ,x_n]}{\sqrt{x_1^2+\cdots +x_n^2}},\cdots ,\frac{[x_{(m-1)n+1},\cdots ,x_{mn}]}{\sqrt{x_1^2+\cdots +x_n^2}}\right] \right\| \\ & =& \left( 1-\frac{1}{\sqrt{m}}\right) \left\| \left[ 1,\cdots ,1\right] \right\| =\sqrt{m}-1~~~~~~~~~~~~~~~~~~~~~~~~~~~~~~~~~~~~~~~~~~~~~~~~~~~~~~~~~~~~~~~~~~~~~~~~~~~~~~~~~~~~~~~~~~~~~~~~~~~~~~~~~~~~~~\square \\ \end{array}$$

Obviously, when the normalization constants of *m* groups are not completely equal, it corresponds to the case of hybrid encoding, while when the normalization constants of *m* groups are completely equal, it corresponds to the case of repetitive amplitude encoding proposed in this paper. The conclusion of the theorem indicates that, in repetitive amplitude encoding, the difference between the probability amplitude and the original data reaches its minimum value, and for a fixed number of groups *m*, the minimum value is unique (i.e. $\sqrt{m}-1$). Hence, the repetitive amplitude encoding has a higher degree of conformity to the original data.

Next, we will verify the theorem numerically. Taking an 8-dimensional random number vector as an example, let $X_1=[x_1, x_2, x_3, x_4, x_5, x_6, x_7, x_8]=[0.6760, 0.6184, 0.8668, 0.3336, 0.3666, 0.1175, 0.0472, 0.9049]$, After $X_1$ is normalized, it becomes $\hat{X}_1=[0.4136, 0.3784, 0.5303, 0.2041, 0.2243, 0.0719, 0.0289, 0.5536]$. Now divide $X_1$ into two groups $\bar{Y}_1=[x_1, x_2, x_3, x_4]$ and $\bar{Y}_2=[x_5, x_6, x_7, x_8]$, and after normalizing the two groups, $\tilde{Y}_1=[0.5182, 0.4740, 0.6644, 0.2557]$, $\tilde{Y}_2=[0.3724, 0.1193, 0.0480, 0.9191]$. Thus, $\hat{Y}_1=[\tilde{Y}_1,\tilde{Y}_2]=$ $[0.5182, 0.4740, 0.6644, 0.2557,$ $0.3724, 0.1193, 0.0480, 0.9191]$. At this time, $\parallel \hat{Y}_1-\hat{X}_1\parallel =0.4459$.

Let $X_2=[x_1, x_2, x_3, x_4, x_1, x_2, x_3, x_4]=$ $[0.6760, 0.6184, 0.8668, 0.3336, 0.6760, 0.6184, 0.8668, 0.3336]$. After $X_2$ is normalized, it becomes $\hat{X}_2=[0.3664, 0.3352, 0.4698,$ $0.1808, 0.3664, 0.3352, 0.4698, 0.1808]$. Obviously $X_2$ contains two identical groups, and after normalizing each group, $X_2$ becomes $\hat{Y}_2=[0.5182, 0.4740, 0.6644,$ $0.2557, 0.5182, 0.4740, 0.6644, 0.2557]$. At this time, $\parallel \hat{Y}_2-\hat{X}_2\parallel =0.4142=\sqrt{2}-1$. Therefore, $\parallel \hat{Y}_2-\hat{X}_2\parallel <\parallel \hat{Y}_1-\hat{X}_1\parallel$, which precisely conforms to the conclusion of the Theorem 1. $\square$

- The repetitive amplitude encoding can expand the sample dimension and introduce nonlinear mapping

For an $n=2^t$ dimensional normalized vector $X=[x_0, x_1, \cdots , x_{2^t-1}]$ that requires *t* qubits of encoding, assuming that the repetitive amplitude encoding uses *m* qubit blocks (each block has *t* qubits). According to the principle of quantum computing, the encoding result is the tensor product of *m* vectors *X*, that is

11 $$\begin{aligned} |q_{1,1}\cdots q_{1,t}\rangle \otimes \cdots \otimes |q_{m,1}\cdots q_{m,t}\rangle =X\otimes \cdots \otimes X=X^{\otimes m}. \end{aligned}$$

It can be seen that the dimension of the encoded vector $X^{\otimes m}$ is $n^m$, which is much larger than the dimension *n* of the original vector *X*. On the other hand, according to the calculation principle of tensor product, the encoding result of repetitive amplitude encoding can be rewritten as

12 $$\begin{aligned} |q_{1,1}\cdots q_{1,t}\rangle \otimes \cdots \otimes |q_{m,1}\cdots q_{m,t}\rangle =\sum \limits _{i_1=0}^{2^t-1}\cdots \sum \limits _{i_m=0}^{2^t-1}x_{i_1}\cdots x_{i_m}|i_1\rangle \otimes \cdots \otimes |i_m\rangle , \end{aligned}$$

where $|i_k\rangle$ is the $i_k$-th computational basis state of the qubits in the *k*-th block, $i_k=0, 1, \cdots , 2^t-1$ and $k=1, 2, \cdots , m$.

According to Eq. (12), the vector after repetitive amplitude encoding contains a total of $(2^t)^m$ numbers, and each number is the product of *m* numbers (possibly duplicates) obtained from the original vector *X*. It is the product of these *m* numbers that introduces the nonlinear mapping of the original sample features.

Below, we provide further explanation on how repetitive amplitude encoding can introduce nonlinear mapping into QNN. Let the state of the quantum register after encoding be $|X\rangle =x_1|0\rangle +x_2|1\rangle +\cdots +x_{2^n}\vert 2^n-1\rangle =[x_1, x_2, \cdots , x_{2^n}]^\textrm{T}$, and the unitary matrix of the parameterized quantum circuit located between encoding and measurement in QNN be represented as $$U=\left[ \begin{array}{cccc}u_{1,1}& u_{1,2}& \cdots & u_{1,2^n}\\ u_{2,1}& u_{2,2}& \cdots & u_{2,2^{n}}\\ \vdots & \vdots & \cdots & \vdots \\ u_{2^n,1}& u_{2^n,2}& \cdots & u_{2^n,2^n}\end{array}\right]$$. After the action of parameterized quantum circuits, the state of the quantum register becomes $$U|X\rangle =\left[ \begin{array}{l}u_{1,1}x_1+u_{1,2}x_2+\cdots +u_{1,2^n}x_{2^n}\\ u_{2,1}x_1+u_{2,2}x_2+\cdots +u_{2,2^n}x_{2^n}\\ \cdots ~~\cdots ~~\cdots ~~\cdots ~~\cdots ~~\cdots ~~\cdots ~~\cdots \\ u_{2^n,1}x_1+u_{2^n,2}x_2+\cdots +u_{2^n,2^n}x_{2^n}\end{array}\right]$$, where each data $u_{i,1}x_1+u_{i,2}x_2+\cdots +u_{i,2^n}x_{2^n} (i=1,2,\cdots ,2^n)$ in $U|X\rangle$ is a linear combination of all data in $|X\rangle$. It can be seen that the nonlinear mapping ability of QNN can only come from subsequent measurements with large randomness.

However, for repetitive amplitude encoding, assuming the number of repetitions is *m*, it can be seen from Eq. (12) that each data $x_{i_1}x_{i_2}\cdots x_{i_m}$ in the encoded quantum register $|X\rangle$ is the product of *m* classical data. Subsequently, these product terms with nonlinear mapping effects are linearly transformed by the parameterized quantum circuit *U* and passed to the output terminal of QNN. After measurement, the output result with nonlinear mapping effects can be obtained. This indicates that the repetitive amplitude encoding method can introduce a non-linear mapping ability to the original sample data formed by the encoding mechanism into QNN.

For the above analysis, we provide the following numerical verification. Let the normalized classical sample data be $X=[x_0, x_1]^\textrm{T}$, and use two qubit blocks (1 qubit per block) to implement repetitive amplitude encoding. At this time, the encoding result is $|q_0\rangle \otimes |q_1\rangle =(x_0|0\rangle +x_1|1\rangle )\otimes$ $(x_0|0\rangle +x_1|1\rangle )=x_0x_0|00\rangle +x_0x_1|01\rangle +$ $x_0x_1|01\rangle +x_1x_0|10\rangle +x_1x_1|11\rangle$ $=[x_0x_0, x_0x_1, x_1x_0, x_1x_1]^\textrm{T}$. Let the matrix of parameterized quantum circuit in QNN be represented as $U=CNOT(R(\theta _1)\otimes R(\theta _2))$. Then when $\theta _1=0.6771$ and $\theta _2=0.3136$,

13 $$\begin{aligned} U(|q_0\rangle \otimes |q_1\rangle )=\left[ \begin{array}{rrrr}0.9317& -0.1473& -0.3281& 0.0519\\ 0.1473& 0.9317& -0.0519& -0.3281\\ 0.0519& 0.3281& 0.1473& 0.9317\\ 0.3281& -0.0519& 0.9317& -0.1473\end{array}\right] \left[ \begin{array}{c}x_0x_0\\ x_0x_1\\ x_1x_0\\ x_1x_1\end{array}\right] =\left[ \begin{array}{c}0.9317x_0x_0-0.1473x_0x_1-0.3281x_1x_0+0.0519x_1x_1\\ 0.1473x_0x_0+0.9317x_0x_1-0.0519x_1x_0-0.3281x_1x_1\\ 0.0519x_0x_0+0.3281x_0x_1+0.1473x_1x_0+0.9317x_1x_1\\ 0.3281x_0x_0-0.0519x_0x_1+0.9317x_1x_0-0.1473x_1x_1\end{array}\right] . \end{aligned}$$

That is, repetitive amplitude encoding enables QNN to achieve the following nonlinear mapping of the original sample data.

14 $$\begin{aligned} \left[ \begin{array}{c}x_0\\ x_1\end{array}\right] \rightarrow \left[ \begin{array}{c}0.9317x_0x_0-0.1473x_0x_1-0.3281x_1x_0+0.0519x_1x_1\\ 0.1473x_0x_0+0.9317x_0x_1-0.0519x_1x_0-0.3281x_1x_1\\ 0.0519x_0x_0+0.3281x_0x_1+0.1473x_1x_0+0.9317x_1x_1\\ 0.3281x_0x_0-0.0519x_0x_1+0.9317x_1x_0-0.1473x_1x_1\end{array}\right] . \end{aligned}$$

From this numerical result, it can be concluded that repetitive amplitude encoding not only expands the dimensionality of the original data, but also introduces nonlinear mapping capability to QNN. From the perspective of statistical learning, the effect of repetitive amplitude encoding is similar to the role of the kernel function in the support vector machine to some extent. By mapping the original linearly inseparable samples to a high-dimensional space, it is possible to make them linearly separable, thereby improving classification performance.

In quantum neural network, a large number of quantum gates can only perform linear transformations, which is currently the main reason for the unsatisfactory mapping ability of the network. Although hybrid encoding can also introduce nonlinear mapping through tensor operations, inconsistent normalization constant affect further improvement of network performance. Therefore, these advantages of repetitive amplitude encoding are crucial for improving the performance of quantum neural network. Subsequent experiments will validate our conclusions.

- The same normalization constant in RAE can improve the fidelity of quantum state representation and reduce the error recognition rate

For classical data $[x_1, x_2, \cdots , x_n]$ and $[y_1, y_2, \cdots , y_n]$, they have different normalization constants. Using amplitude encoding, they can be encoded into the probability amplitudes of *n* qubits $q_1, q_2, \cdots , q_n$ and $q_{n+1}, q_{n+2}, \cdots , q_{2n}$, respectively.

For any $\lambda _x > 0$, all vectors $\lambda _x[x_1, x_2, \cdots , x_n]$ after normalization are identical, and thus the encoded quantum states are also the same. Similarly, for any $\lambda _y > 0$, all vectors $\lambda _y[y_1, y_2, \cdots , y_n]$ after normalization are identical, and the encoded quantum states remain the same. Therefore, for any $\lambda _x > 0$ and any $\lambda _y > 0$, a QNN using hybrid encoding will be unable to distinguish between the quantum states encoded from $\lambda _x[x_1, x_2, ..., x_n]$ and $\lambda _y[y_1, y_2, \cdots , y_n]$. The number of classical data that these quantum states cannot distinguish depends on two degrees of freedom $(\lambda _x, \lambda _y)$.

For RAE, the two sets of qubits use the same classical data encoding, and the normalization constants of the two datasets are identical. In this case, although for any $\lambda _x > 0$, a QNN using RAE still cannot distinguish between the quantum states encoded from $\lambda _x[x_1, x_2, \cdots , x_n]$ and $\lambda _x[x_1, x_2, \cdots , x_n]$, the number of classical data that these quantum states cannot distinguish depends only on a single degree of freedom $\lambda _x$, which is far fewer than in the case of hybrid encoding. This is the fundamental reason why the same normalization constant in RAE can improve the fidelity of quantum state representation and reduce the error recognition rate.

#### A structured theoretical comparison of RAE versus amplitude encoding, hybrid encoding, and angle encoding

- Comparison under the same number of data.

For *N* classical data, the number of qubits required for amplitude encoding is $\log _2(N)$. Obviously, with amplitude encoding, a quantum computer can represent exponentially many classical data. However, the quantum circuit depth for amplitude encoding usually grows as $O(\textrm{poly}(N))$. Angle encoding requires up to *N* qubits, but the depth of the circuit remains constant. For hybrid encoding, assuming *N* data are divided into *b* groups, the required number of qubits is $b\log _2(N/b)$, and the depth of the encoding circuit is $O(\textrm{poly}(N/b))$. For repetitive amplitude encoding, assuming *b* repetitions, the required number of qubits is $b\log _2(N)$, and the depth of the encoding circuit is $O(\textrm{poly}(N))$.

- Comparison under the same number of qubits

For an *n*-bit quantum register, the data capacity of amplitude encoding is $N=2^n$, and the circuit depth is $O(\textrm{poly}(N))$. Angle encoding can only encode $N=n$ classical data, but the circuit depth is constant. For hybrid encoding, assuming that *n* qubits are divided into *b* groups, the data capacity is $N=b2^{n/b}$, and the depth of the encoding circuit is $O(\textrm{poly}(N/b))$. For repetitive amplitude encoding, assuming the number of repetitions is *b*, at this time, $N=2^{n/b}$ classical data can be encoded, and the depth of the encoding circuit is $O(\textrm{poly}(N/b))$.

#### Implementation constraints for repetitive amplitude encoding

Applying repetitive amplitude encoding, a potential constraint is that the dimensionality of the sample must satisfy an integer power of 2. In fact, this constraint is not unique to repetitive amplitude encoding, and is also present in amplitude encoding and hybrid encoding. For samples whose dimensions do not meet this constraint, the dimensions can be adjusted to meet the constraint through dimensionality reduction, interpolation, or zero padding. The computational complexity of repetitive amplitude encoding is also identical to amplitude encoding and hybrid encoding with the same number of qubits.

### QNN classification model using repetitive amplitude encoding

#### Ansatz

**Fig. 2:**
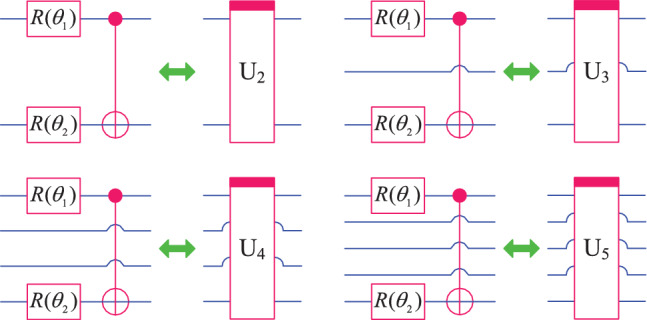
Four forms of ansatz used to construct QNN.

In general, the QNN structure is flexible and can use any two qubit unitary operation in each cascaded layer. In this paper, we stipulate that all layers of QNN use the same ansatz. Regarding the selection of ansatz, 9 different structures are presented in^34^, all of which are composed of single qubit rotation gates and 2-qubit controlled gates, but there are significant differences in circuit depth. The superiority of QNN depends on the expressivity of the employed ansatze. However, relevant research results show that as the depth of quantum circuits increases, the expressibility of quantum neural network decreases exponentially^41^, and the probability of the appearance of barren plateaus increases significantly^42^. Therefore, this paper adopts ansatz with relatively shallow depth as the basic unit for constructing QNN. These ansatzs have only two optimization parameters, but they can be applied to 2, 3, 4, and 5 qubits respectively. The specific quantum circuit and symbol are shown in Fig. 2.

The matrix representation of the unitary operators corresponding to these ansatz are as follows.

15 $$\begin{aligned} \left\{ \begin{array}{lll} U_2 & = & (I\oplus X)\times (R(\theta _1)\otimes R(\theta _2)), \\ U_3 & = & (I^{\otimes 2}\oplus (I\otimes X))\times (R(\theta _1)\otimes I\otimes R(\theta _2)), \\ U_4 & = & (I^{\otimes 3}\oplus (I^{\otimes 2}\otimes X))\times (R(\theta _1)\otimes I^{\otimes 2}\otimes R(\theta _2)), \\ U_5 & = & (I^{\otimes 4}\oplus (I^{\otimes 3}\otimes X))\times (R(\theta _1)\otimes I^{\otimes 3}\otimes R(\theta _2)). \end{array} \right. \end{aligned}$$

#### QNN classification model

**Fig. 3:**
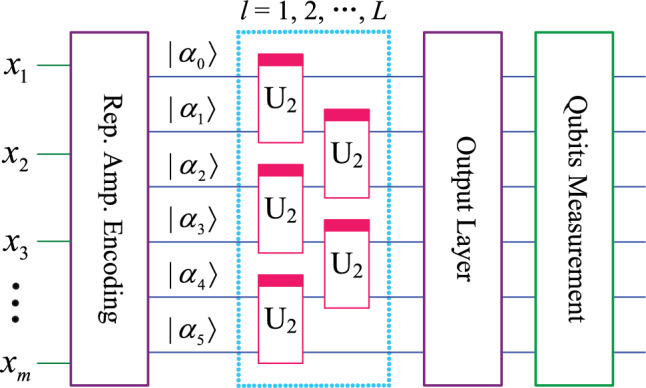
QNN model that can classify up to six classes.

**Fig. 4:**
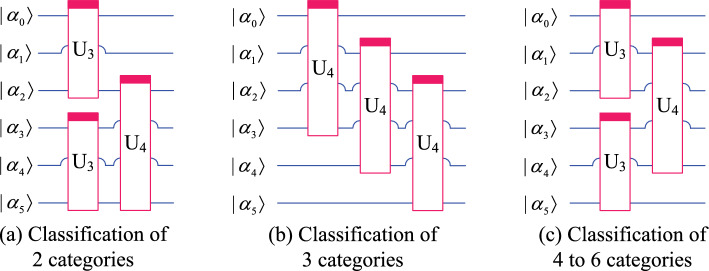
Output layer circuits for different number of classes.

**Fig. 5:**
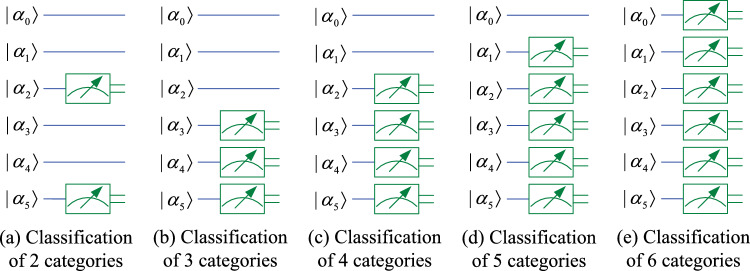
Qubits measured for different number of classes.

Using the ansatz introduced earlier in this paper, the QNN used for classification of 2 to 6 classes is shown in Fig. 3. In this model, a total of 6 qubits $(\alpha _0, \alpha _1, \cdots , \alpha _5)$ are used. The module on the far left performs the encoding of *m* classical data, which is equivalent to the input layer of the classical neural network. Next, the 6-qubits quantum system is continuously acted on by a parameterized quantum circuit (also called hidden layer) composed of 5 ansatz for *l* times and then submitted to the output layer. The output layer is also a parameterized quantum circuit. After further processing by the output layer, the quantum system can be measured to obtain classical output results. Output layer circuits for different number of classes are shown in Fig. 4, and qubits measured for different number of classes are shown in Fig. 5.

According to Figs. 3 and 4, for classification of 2 to 6 classes, the mapping relationship of parameterized quantum circuits in QNN can be written as

16 $$\begin{aligned} \left\{ \begin{array}{lll} map_2 & = & (I^{\otimes 2}\otimes U_4)U_3^{\otimes 2}((I\otimes U_2^{\otimes 2}\otimes I)U_2^{\otimes 3})^l, \\ map_3 & = & (I^{\otimes 2}\otimes U_4)(I\otimes U_4\otimes I)(U_4\otimes I^{\otimes 2})((I\otimes U_2^{\otimes 2}\otimes I)U_2^{\otimes 3})^l, \\ map_{4,5,6} & = & (I\otimes U_4\otimes I)U_3^{\otimes 2}((I\otimes U_2^{\otimes 2}\otimes I)U_2^{\otimes 3})^l. \end{array} \right. \end{aligned}$$

In the QNN shown in Fig. 3, the parameterized quantum circuit has 6 qubits $(|\alpha _0\rangle , |\alpha _1\rangle , |\alpha _2\rangle , |\alpha _3\rangle , |\alpha _4\rangle , |\alpha _5\rangle )$. According to the repetitive amplitude encoding, if these 6 qubits are divided into 2 groups, such as $(|\alpha _0\rangle , |\alpha _1\rangle , |\alpha _2\rangle )$ and $(|\alpha _3\rangle , |\alpha _4\rangle , |\alpha _5\rangle )$, then these qubits can encode 8 normalized classical data $(x_0, x_1, x_2, x_3, x_4, x_5, x_6, x_7)$. The repetitive amplitude encoding result is as follows.

17 $$\begin{aligned} \left( \sum \limits _{i=0}^{7}x_i|i\rangle \right) \left( \sum \limits _{j=0}^{7}x_j|j\rangle \right) =\sum \limits _{i=0}^{7}\sum \limits _{j=0}^{7}x_ix_j|ij\rangle , \end{aligned}$$

where $|i\rangle$ is the basis state of the quantum superposition state $|\alpha _0\alpha _1\alpha _2\rangle$, and $|j\rangle$ is the basis state of $|\alpha _3\alpha _4\alpha _5\rangle$.

If these 6 qubits are divided into 3 groups, such as $$(|\alpha _0\rangle , |\alpha _1\rangle )$$, $$(|\alpha _2\rangle , |\alpha _3\rangle )$$, and $$(|\alpha _4\rangle , |\alpha _5\rangle )$$, then these qubits can encode 4 normalized classical data $$(x_0, x_1$$, $$x_2, x_3)$$. The encoding result is as follows.

18 $$\begin{aligned} \left( \sum \limits _{i=0}^{3}x_i|i\rangle \right) \left( \sum \limits _{j=0}^{3}x_j|j\rangle \right) \left( \sum \limits _{k=0}^{3}x_k|k\rangle \right) =\sum \limits _{i=0}^{3}\sum \limits _{j=0}^{3}\sum \limits _{k=0}^{3}x_ix_jx_k|ijk\rangle , \end{aligned}$$

where $|i\rangle , |j\rangle$, and $|k\rangle$ are the basis state of the quantum superposition state $|\alpha _0\alpha _1\rangle , |\alpha _2\alpha _3\rangle$, and $|\alpha _4\alpha _5\rangle$, respectively.

**Fig. 6:**
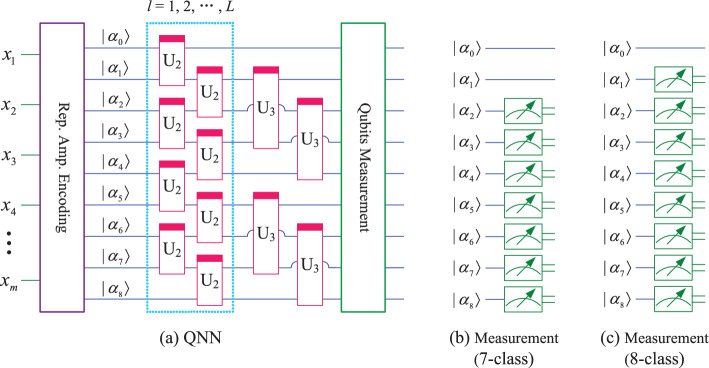
QNN model for 7−8 classification.

**Fig. 7:**
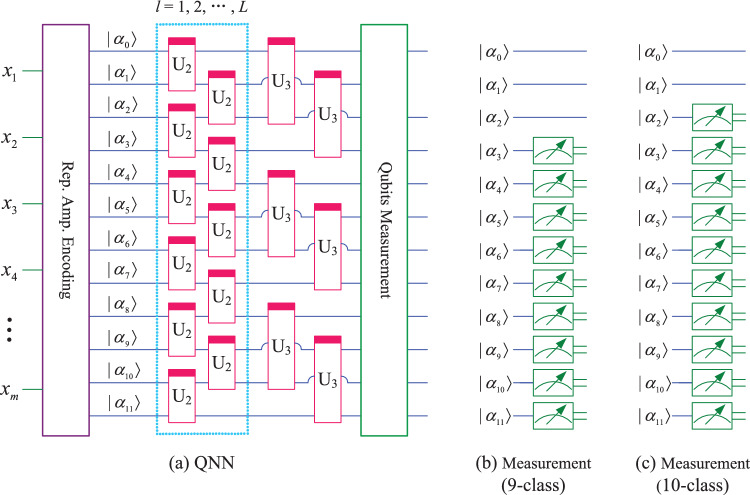
QNN model for $9-10$ classification.

Similar to Figs. 3, 4 and 5, for classification of 7 to 8 classes, the QNN designed in this paper uses 9 qubits, as shown in Fig. 6. Similarly, for classification of 9 to 10 classes, the QNN uses 12 qubits, as shown in Fig. 7. In Figs. 6 and 7, subfigure (a) represents the QNN model, while subfigures (b, c) show the qubits measured for different number of class.

the mapping relationship of parameterized quantum circuits in QNN can be written separately as

19 $$\begin{aligned} \left\{ \begin{array}{lll} map_{7,8} & = & (I\otimes (I\otimes U_3)^{\otimes 2})((I\otimes U_3)^{\otimes 2}\otimes I)((I\otimes U_2^{\otimes 4})(U_2^{\otimes 4}\otimes I))^l, \\ map_{9,10} & = & (I\otimes U_3)^{\otimes 3}\otimes (U_3\otimes I)^{\otimes 3}((I\otimes U_2^{\otimes 5}\otimes I)U_2^{\otimes 6})^l. \end{array} \right. \end{aligned}$$

In the QNN shown in Fig. 6, the parameterized quantum circuit has 9 qubits $(|\alpha _0\rangle , |\alpha _1\rangle , |\alpha _2\rangle , |\alpha _3\rangle , |\alpha _4\rangle , |\alpha _5\rangle , |\alpha _6\rangle , |\alpha _7\rangle , |\alpha _8\rangle )$. These 9 qubits can be divided into 3 groups, such as $(|\alpha _0\rangle , |\alpha _1\rangle , |\alpha _2\rangle )$, $(|\alpha _3\rangle , |\alpha _4\rangle , |\alpha _5\rangle )$, and $(|\alpha _6\rangle , |\alpha _7\rangle , |\alpha _8\rangle )$, then these qubits can encode 8 normalized classical data $(x_0, x_1, x_2, x_3, x_4, x_5, x_6, x_7)$. The repetitive amplitude encoding result is as follows.

20 $$\begin{aligned} \left( \sum \limits _{i=0}^{7}x_i|i\rangle \right) \left( \sum \limits _{j=0}^{7}x_j|j\rangle \right) \left( \sum \limits _{k=0}^{7}x_k|k\rangle \right) =\sum \limits _{i=0}^{7}\sum \limits _{j=0}^{7}\sum \limits _{k=0}^{7}x_ix_jx_k|ijk\rangle , \end{aligned}$$

where $|i\rangle , |j\rangle$, and $|k\rangle$ are the basis state of the quantum superposition state $|\alpha _0\alpha _1\alpha _2\rangle , |\alpha _3\alpha _4\alpha _5\rangle$, and $|\alpha _6\alpha _7\alpha _8\rangle$, respectively.

We can also use only the last 8 qubits $\alpha _1, \alpha _2, \alpha _3, \alpha _4, \alpha _5, \alpha _6, \alpha _7, \alpha _8$, which can be divided into 2 groups $(\alpha _1, \alpha _2, \alpha _3, \alpha _4)$, and $(\alpha _5, \alpha _6, \alpha _7, \alpha _8)$. Each group can encode 16 normalized classical data $(x_0, x_1, \cdots , x_{15})$, and the repetitive amplitude encoding result can be written as

21 $$\begin{aligned} \left( \sum \limits _{i=0}^{15}x_i|i\rangle \right) \left( \sum \limits _{j=0}^{15}x_j|j\rangle \right) =\sum \limits _{i=0}^{15}\sum \limits _{j=0}^{15}x_ix_j|ij\rangle , \end{aligned}$$

where $|i\rangle$, and $|j\rangle$ are the basis state of the quantum superposition state $|\alpha _1\alpha _2\alpha _3\alpha _4\rangle$, and $|\alpha _5\alpha _6\alpha _7\alpha _8\rangle$, respectively.

Similarly, the QNN in Fig. 7 use 12 qubits. If these qubits are divided into 2 groups of 6 qubits each, 64 normalized classical data $(x_0, x_2, \cdots , x_{63})$ can be encoded. If these qubits are divided into 3 groups of 4 qubits each, 16 normalized classical data $(x_0, x_2, \cdots , x_{15})$ can be encoded. If these qubits are divided into 4 groups of 3 qubits each, 8 normalized classical data $(x_0, x_2, \cdots , x_7)$ can be encoded. The results of repetitive amplitude encoding for three scenarios can be written separately as

22 $$\begin{aligned} \left\{ \begin{array}{l} \left( \sum \limits _{i=0}^{63}x_i|i\rangle \right) \left( \sum \limits _{j=0}^{63}x_j|j\rangle \right) =\sum \limits _{i=0}^{63}\sum \limits _{j=0}^{63}x_ix_j|ij\rangle ,\\ \left( \sum \limits _{i=0}^{15}x_i|i\rangle \right) \left( \sum \limits _{j=0}^{15}x_j|j\rangle \right) \left( \sum \limits _{k=0}^{15}x_k|k\rangle \right) =\sum \limits _{i=0}^{15}\sum \limits _{j=0}^{15}\sum \limits _{k=0}^{15}x_ix_jx_k|ijk\rangle ,\\ \left( \sum \limits _{i=0}^{7}x_i|i\rangle \right) \left( \sum \limits _{j=0}^{7}x_j|j\rangle \right) \left( \sum \limits _{k=0}^{7}x_k|k\rangle \right) \left( \sum \limits _{l=0}^{7}x_l|l\rangle \right) =\sum \limits _{i=0}^{7}\sum \limits _{j=0}^{7}\sum \limits _{k=0}^{7}\sum \limits _{l=0}^{7}x_ix_jx_kx_l|ijkl\rangle . \end{array} \right. \end{aligned}$$

From Eqs. (17, 18, 20-22), it can be seen that the repetitive amplitude encoding uses multiple qubit blocks to encode the same classical data. By utilizing the superposition and entanglement of multiple qubit blocks, QNN enhance their nonlinear mapping ability to classical data.

It is worth noting that although the QNN shown in Figs. 6 and 7 can also be used for classification of 2 to 6 classes, the large number of qubits used will result in a plateau in the loss function during the training process^16^, which will affect the classification performance. So in this paper, the number of qubits used for classification with fewer classes (i.e. 2,3,4,5,6) is also relatively small.

#### Training of QNN model

Similar to classical neural network training, the first step is to define a loss function. *Cross* *entropy* loss can measure the performance of classification models and is widely used in training classical neural network. Its output is a probability in the interval (0, 1). For QNN, the *cross* *entropy* loss can also be calculated by measuring the probability that a single qubit is in the basis state $|1\rangle$. Taking the *i*th training data as an example, the *cross* *entropy* loss can be expressed as

23 $$\begin{aligned} C(\theta )=-\sum \limits _{c=1}^{C}y_{i,c}\log (\textrm{Pr}[\psi _{ c}(\theta )={ y_{i,c}}]), \end{aligned}$$

where *C* is the number of classes, $y_{i,c}\in \{0, 1\}$ is the label and $\textrm{Pr}[\psi _{ c}(\theta )={ y_{i,c}}]$ is the probability of measuring the computational basis state $|y_{i,c}\rangle$ from the QNN circuit.

The optimization of the quantum gate parameters can be carried out by iteratively updating the parameters based on the gradient of the cost function until some condition for the termination is reached. The gradient of the loss function with respect to optimizing parameters can be calculated using either the chain differentiation rule or parameter-shift rule^43^. Taking binary classification as an example, the parameter adjustment method based on a single training sample see Appendix A in the Supplementary Material. In this paper, we uses the Python software package provided by the PennyLane platform to train QNN, which is based on parameter-shift rule. The specific training process is shown in in Algorithm 1. To highlight the key points, this algorithm omits the encoding of classical data.

**Algorithm 1:**
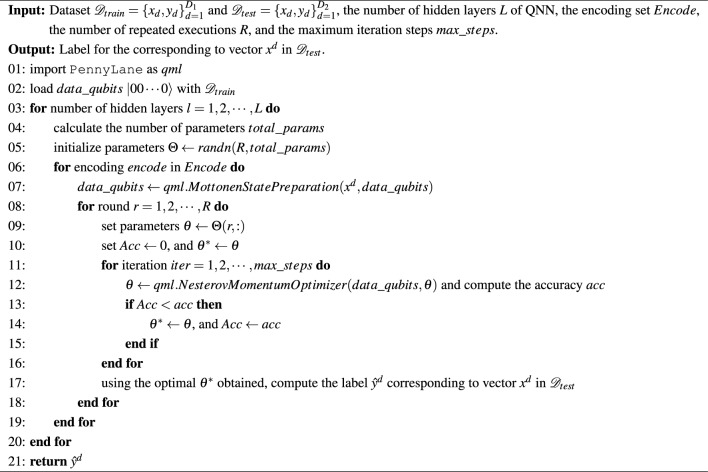
Training of QNN classification model.

This algorithm consists of four loops: first, according to the number of hidden layers, then according to the encoding method, then according to the specified number of rounds, and finally according to the iteration steps. We place the initialization of the parameter $\theta$ in the outermost layer, which ensures that different encoding methods and different rounds are executed based on the same initial parameter value, thus ensuring fairness in the comparison of different encoding methods.

## Results

In this section, we present our experimental results. Since general-purpose quantum computers are not yet widely available, all experiments are performed on classical computers. The entire variational quantum circuits are implemented using the “qml.qnode” simulator provided by PennyLane. The optimization of parameterized quantum circuits is very complex, but fortunately PennyLane provides the necessary Python package. Therefore, we can use quantum operations written in the PennyLane package on classical computers to perform optimization of parameterized quantum circuits. The following example code snippet describes the interface for calling a software package.
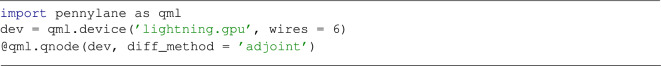


The method qml.device is used to initialize a quantum device or a quantum simulator for performing quantum operations with the number of qubits given as input to wires. lightning.gpu indicates that qml.device can utilize the NVIDIA cuQuantum library under-the-hood, and includes efficient computation of quantum gradients via adjoint differentiation. Use lightning.gpu for a significant speed-up for large quantum circuit evaluations, even when using multiple CPU-threads. The decorator @qml.qnode(dev, diff_method) is used for creating a quantum node on the simulator. Gates in the variational circuit are implemented using qml.RY operations and the entanglement between the qubits are created using qml.CNOT operation. The Measurement operation on the qubits is performed on each wire using qml.exp.PauliZ(n), where $n = 1, 2, \cdots , k$ is the label of the wire. The output values from the measurement are passed through Eq. (23). The model is trained and the gate parameters are optimized classically using the optimization techniques NesterovMomentumOptimizer provided in the package pennylane.optimze. PennyLane provides necessary interfaces to perform hybrid classical-quantum operations. The details of the results for the simulations along with discussions are given in the following sections.

### Evaluation metrics

The evaluation metrics used in this paper to evaluate the classification results include *Accuracy*, *Precision*, *Recall*, *F1-score*, etc. Their specific definitions are as follows.

24 $$\begin{aligned} { Accuracy} = \frac{{ TP+TN}}{{ TP+FP+FN+TN},} { Precision} = \frac{{ TP}}{{ TP+FP}}, { Recall} = \frac{{ TP}}{{ TP+FN}}. \end{aligned}$$

Among them, *TP*, *TN*, *FP*, and *FN* respectively represent the number of true positive, true negative, false positive, and false negative samples in the recognition results.

*F1-score* is defined as the harmonic mean between *Precision* and *Recall*. Its use facilitates the processing of dataset with highly disproportionately diverse classes.

25 $$\begin{aligned} { F1}\!-\!{ score} = \frac{2\times Precision\times Recall}{Precision+Recall}. \end{aligned}$$

In addition, the confusion matrix, the *ROC* curves and corresponding *AUC* values are also used.

### Experimental results on handwritten digit dataset MNIST

All samples in the MNIST dataset are $28\times 28$ images with ten classes. The number of samples of each digit (from 0 to 9) is: [5923, 6742, 5958, 6131, 5842, 5421, 5918, 6265, 5851, 5949] for the training set, and [980, 1135, 1032, 1010, 982, 892, 958, 1028, 974, 1009] for testing set.

The preprocessing mainly focuses on dimensionality reduction operations for the MNIST dataset. Due to the noise and technical challenges of building quantum hardware, the size of quantum circuits that can be reliably executed on Noisy Intermediate-Scale Quantum (NISQ) devices is limited. As the original data is $28\times 28$ image, the number of qubits required for encoding high-dimensional image data usually exceeds the capabilities of current quantum devices, it must be subjected to dimensionality reduction processing to make it suitable for encoding in current quantum systems. In this work, the dimensionality reduction schemes we adopted are autoencoding (AutoEnc)^44^ and bilinear interpolation, both of which are currently the most popular and widely used methods.

#### Parameter settings

This experiment is performed according to Algorithm 1. For each iteration of the training process, we first randomly select 100*N* (*N* is the number of classes) of data from the training set, and then use the small batch method to train the network parameters based on the selected samples, where the batch size is 50. Compared to training on a complete dataset, training on small batches not only reduces simulation time but also helps gradients escape local minima. To demonstrate the fairness of the experimental results, in this experiment, all samples (rather than a subset) of each class in the test set are used for testing to evaluate the performance of different encoding methods. The learning rate of PennyLane optimizer is set to 0.01, and the number of iteration steps is set to 100. To reduce the randomness of optimization results, the network is trained 5 rounds based on different initial values of variational quantum circuit parameters, and the average of the five results is used as the evaluation and comparison. The detailed parameter settings are shown in Tab. 1.

**Table 1:** Detailed settings of QNN related parameters.

Sample set	QNN
Training set:	Software package:
Number of samples per class: 100	pennylane as qml
Selection method: Random	Encoding implementation:
Total number of samples:	qml.templates.embeddings.AmplitudeEmbedding
100*N* (*N* is the number of classes)	qml.templates.embeddings.AngleEmbedding
Batch: $batch=50$	qml.templates.state_preparations.MottonenStatePreparation
Test set:	optimizer: qml.NesterovMomentumOptimizer
Number of samples per class: All	Loss function: Cross entropy
Dimensionality reduction:	Number of hidden layers: $k=1, 2, 3, 4$
Autoencoder	Learning rate: 0.01
Autoencoder parameter:	Iteration steps: 100
optimizer=’adam’	Number of independent runs: $round=5$
loss=MeanSquaredError	Number of parameters (*k* is the number of hidden layers):
epochs=10	$total=10k+6~~$ for $2-6$ classes
shuffle=True	$total=16k+8~~$ for $7-8$ classes
	$total=22k+12~~$ for $9-10$ classes
	Initialization method: np.random.randn(*round*, *total*)

#### Comparison of different encoding methods from 2 to 6 classes

To verify the advantages of repetitive amplitude encoding, we compared it in detail with hybrid encoding, angle encoding, and amplitude encoding based on QNN in Fig. 3. At this point, the parameterized quantum circuits has 6 qubits, which we divide into two groups $(|\alpha _0\rangle , |\alpha _1\rangle , |\alpha _2\rangle )$ and $(|\alpha _3\rangle , |\alpha _4\rangle , |\alpha _5\rangle )$. Therefore, the number of classical data that can be encoded by repetitive amplitude encoding, hybrid encoding, angle encoding, and amplitude encoding are 8, 16, 6, and 64, respectively. For amplitude encoding, bilinear interpolation is used for dimensionality reduction, and the other three encodings use autoencoders for dimensionality reduction. For ease of description, in the subsequent figures and tables, the encoding methods are abbreviated as Re*n* (repetitive amplitude encoding of *n* qubit blocks), Hyb (hybrid encoding), Ang (angle encoding), and Amp (amplitude encoding). To fully investigate the influence of the depth of parameterized quantum circuits on encoding performance, we set the number of hidden layers in QNN to $l=1,2,3,4$.

For experiments with different numbers of classes, four datasets were selected according to different digits combinations. Specifically, two classes: [0, 1], [3, 4], [6, 7], [9, 0]; three classes: [0, 3, 6], [1, 4, 7], [2, 5, 8], [3, 6, 9]; four classes: [1, 2, 3, 4], [1, 3, 5, 7], [2, 4, 6, 8], [5, 6, 7, 8]; five classes: [1, 2, 3, 4, 5], [1, 3, 5, 7, 9], [2, 4, 6, 8, 0], [6, 7, 8, 9, 0]; and six classes: [1, 2, 3, 4, 5, 6], [2, 3, 4, 5, 6, 7], [3, 4, 5, 6, 7, 8], [4, 5, 6, 7, 8, 9].

For each data set, QNN is trained 5 times independently for each combination of encoding method and number of hidden layers, and then compared with the average value of the evaluation metrics. The *Accuracy* of 2 to 4 classes on their respective test sets is shown in Table 2, and the *Accuracy* of 5 to 6 classes on their respective test sets is shown in Table 3.

**Table 2:** Comparison of *Accuracy* under different encodings for the 2 to 4 classes test set. The results shown are the mean *Accuracy* of the test set for 5 independent training. The number with the highest *Accuracy* among the all encoding methods is represented in bold.

Number of class	Test set	Encoding	1 Hidden layer	2 Hidden layers	3 Hidden layers	4 Hidden layers
2	[0,1]	Re2	**0.9971**	**0.9969**	**0.9965**	**0.9975**
		Hyb	0.9884	0.9963	0.9957	0.9967
		Ang	0.9964	0.9958	0.9955	0.9938
		Amp	0.9850	0.9855	0.9854	0.9876
	[3,4]	Re2	**0.9830**	**0.9857**	**0.9884**	**0.9892**
		Hyb	0.9411	0.9697	0.9836	0.9800
		Ang	0.9532	0.9854	0.9880	0.9848
		Amp	0.9229	0.9542	0.9546	0.9587
	[6,7]	Re2	**0.9823**	**0.9895**	**0.9911**	**0.9923**
		Hyb	0.9679	0.9763	0.9841	0.9843
		Ang	0.9757	0.9886	0.9884	0.9879
		Amp	0.8906	0.9069	0.9185	0.9294
	[9,0]	Re2	**0.9751**	0.9783	0.9804	**0.9839**
		Hyb	0.9078	**0.9813**	**0.9845**	0.9824
		Ang	0.9210	0.9783	0.9821	0.9791
		Amp	0.9484	0.9580	0.9671	0.9721
3	[0,3,6]	Re2	0.8881	**0.9379**	**0.9463**	**0.9477**
		Hyb	0.8773	0.9015	0.9277	0.9369
		Ang	**0.9118**	0.9136	0.9341	0.9408
		Amp	0.8069	0.8506	0.8592	0.8718
	[1,4,7]	Re2	**0.9228**	**0.9474**	**0.9568**	**0.9643**
		Hyb	0.8659	0.8885	0.9325	0.9447
		Ang	0.8478	0.8950	0.9202	0.9175
		Amp	0.7793	0.7933	0.8176	0.8324
	[2,5,8]	Re2	**0.8376**	**0.8622**	**0.8825**	**0.8888**
		Hyb	0.7039	0.8009	0.8337	0.8533
		Ang	0.7876	0.8297	0.8556	0.8528
		Amp	0.7325	0.7340	0.7549	0.7604
	[3,6,9]	Re2	**0.9212**	**0.9431**	**0.9516**	0.9552
		Hyb	0.9053	0.9412	0.9431	**0.9559**
		Ang	0.8107	0.9261	0.9409	0.9464
		Amp	0.7414	0.7922	0.8118	0.8144
4	[1,2,3,4]	Re2	**0.8938**	**0.9258**	**0.9192**	**0.9281**
		Hyb	0.7048	0.8033	0.8690	0.8878
		Ang	0.6554	0.7132	0.8297	0.8718
		Amp	0.6578	0.7071	0.7592	0.7510
	[1,3,5,7]	Re2	0.7982	**0.8461**	**0.8751**	**0.8847**
		Hyb	**0.8106**	0.8153	0.8589	0.8695
		Ang	0.7381	0.8202	0.8492	0.8508
		Amp	0.6301	0.7167	0.7529	0.7531
	[2,4,6,8]	Re2	**0.8010**	**0.8555**	**0.8944**	**0.9024**
		Hyb	0.7383	0.8112	0.8488	0.8557
		Ang	0.6293	0.7666	0.8431	0.8774
		Amp	0.4803	0.5744	0.6234	0.6496
	[5,6,7,8]	Re2	**0.8178**	**0.8483**	**0.8710**	**0.8860**
		Hyb	0.7362	0.7830	0.8155	0.8296
		Ang	0.6905	0.7727	0.7864	0.8070
		Amp	0.5633	0.6286	0.6670	0.6861

**Table 3:** Comparison of *Accuracy* under different encodings for the 5 and 6 classes test set. The results shown are the mean *Accuracy* of the test set for 5 independent training. The number with the highest *Accuracy* among the all encoding methods is represented in bold.

Number of class	Test set	Encode	1 hidden layer	2 hidden layers	3 hidden layers	4 hidden layers
5	[1,2,3,4,5]	Re2	**0.6802**	**0.7678**	**0.8204**	**0.8306**
		Hyb	0.6318	0.7036	0.7581	0.8010
		Ang	0.5269	0.6765	0.7082	0.7690
		Amp	0.5132	0.5833	0.6316	0.6248
	[1,3,5,7,9]	Re2	0.7021	**0.7409**	**0.7808**	**0.7933**
		Hyb	**0.7031**	0.6860	0.7651	0.7700
		Ang	0.6080	0.6514	0.7217	0.7402
		Amp	0.5818	0.6146	0.6487	0.6512
	[2,4,6,8,0]	Re2	**0.7133**	**0.8290**	**0.8443**	**0.8629**
		Hyb	0.5970	0.6757	0.7660	0.8161
		Ang	0.6114	0.6955	0.7707	0.7981
		Amp	0.4887	0.5981	0.5954	0.6475
	[6,7,8,9,0]	Re2	**0.7245**	**0.8092**	**0.8489**	**0.8563**
		Hyb	0.6336	0.7450	0.7955	0.8239
		Ang	0.6038	0.6651	0.7445	0.7747
		Amp	0.4706	0.6440	0.6660	0.6749
6	[1,2,3,4,5,6]	Re2	**0.6846**	**0.7797**	**0.7911**	**0.8157**
		Hyb	0.5930	0.7395	0.7709	0.8092
		Ang	0.4971	0.6012	0.6506	0.7025
		Amp	0.4494	0.5196	0.5241	0.5387
	[2,3,4,5,6,7]	Re2	**0.6475**	**0.7364**	**0.7825**	**0.8231**
		Hyb	0.5453	0.6528	0.7221	0.7578
		Ang	0.5615	0.6182	0.6923	0.7080
		Amp	0.3917	0.4884	0.5130	0.5483
	[3,4,5,6,7,8]	Re2	**0.6518**	**0.7191**	**0.7372**	**0.7681**
		Hyb	0.4241	0.6255	0.6584	0.7011
		Ang	0.4500	0.5502	0.6124	0.6627
		Amp	0.4664	0.5065	0.5496	0.5660
	[4,5,6,7,8,9]	Re2	**0.6208**	**0.6772**	**0.6941**	**0.7243**
		Hyb	0.5756	0.6370	0.6695	0.6806
		Ang	0.4019	0.5410	0.6305	0.6361
		Amp	0.4449	0.4734	0.5291	0.5313

The results shown in Tables 2 and 3 indicate that, in all experiments, the *Accuracy* of repetitive amplitude encoding is not only generally higher than that of the other three encodings, but also the degree of its superiority increases with the increase of the number of classes. This suggests that repetitive amplitude encoding has more potential for handling complex classification problems.

#### Comparison of different encoding methods for 7 to 10 classes

**Fig. 8:**
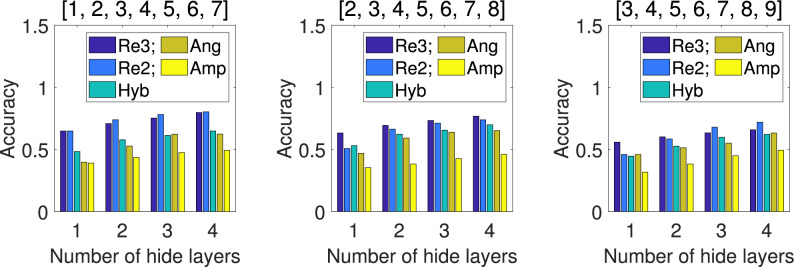
Comparison of *Accuracy* under different encodings for the 7-class test set. The results shown are the mean *Accuracy* of 5 independent QNN training.

**Fig. 9:**
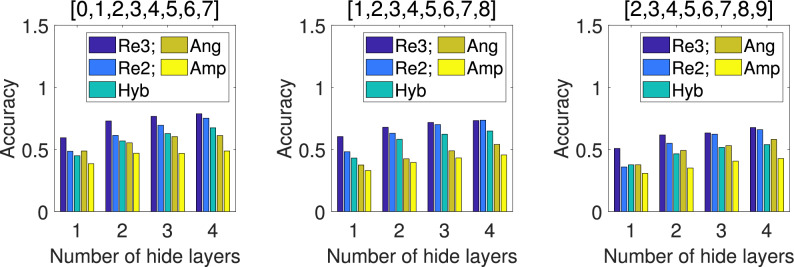
Comparison of *Accuracy* under different encodings for the 8-class test set. The results shown are the mean *Accuracy* of 5 independent QNN training.

For the 7-class and 8-class classification, we select 3 sample sets based on combinations of different digits. At this point, the 6-qubit QNN is no longer suitable, so we use the 9-qubit model shown in Fig. 6 instead. For repetitive amplitude encoding, we divide the 9 qubits into 3 groups $(|\alpha _0\rangle , |\alpha _1\rangle , |\alpha _2\rangle )$, $(|\alpha _3\rangle , |\alpha _4\rangle , |\alpha _5\rangle )$, and $(|\alpha _6\rangle , |\alpha _7\rangle , |\alpha _8\rangle )$, which can encode 8 classical data. We can also use only the last 8 qubits and divide them into 2 groups $(|\alpha _1\rangle , |\alpha _2\rangle , |\alpha _3\rangle , |\alpha _4\rangle )$ and $(|\alpha _5\rangle , |\alpha _6\rangle , |\alpha _7\rangle , |\alpha _8\rangle )$, which can encode 16 classical data. For hybrid encoding, angle encoding, and amplitude encoding, with only the last 8 qubits, they can encode 32, 8, and 256 classical data, respectively. The comparison schemes are the same as above. The mean *Accuracy* of 5 independent training under different encoding methods for the test set are shown in Figs. 8 and 9, and the mean *F1-score* of 5 independent training under each encoding for the test set are shown in Table 4.

**Table 4:** Comparison of *F1-score* under different encodings for the 7 and 8 classes test set. The results shown are the mean *F1-score* of the test set for 5 independent training. The number with the highest *Accuracy* among the all encoding methods is represented in bold.

Number of class	Test set	Encode	1 hidden layer	2 hidden layers	3 hidden layers	4 hidden layers
7		Re3	**0.6354**	0.6699	0.7454	0.7952
		Re2	0.6313	**0.7283**	**0.7749**	**0.6**
	[1,2,3,4,5,6,7]	Hyb	0.4509	0.5500	0.6031	0.6412
		Ang	0.3557	0.5004	0.5971	0.5978
		Amp	0.2998	0.3839	0.4186	0.4392
		Re3	**0.6060**	**0.6787**	**0.7162**	**0.7654**
		Re2	0.4839	0.6505	0.7047	0.7342
	[2,3,4,5,6,7,8]	Hyb	0.4968	0.6078	0.6463	0.7004
		Ang	0.4313	0.5691	0.6154	0.6258
		Amp	0.2535	0.3083	0.3848	0.4276
		Re3	**0.5555**	**0.5757**	0.6157	0.6317
		Re2	0.4278	0.5655	**0.6751**	**0.7148**
	[3,4,5,6,7,8,9]	Hyb	0.4067	0.5116	0.5812	0.6119
		Ang	0.4420	0.5045	0.5336	0.6234
		Amp	0.2545	0.3260	0.4184	0.4649
8		Re3	**0.5665**	**0.7106**	**0.7561**	**0.7801**
		Re2	0.4564	0.5843	0.6750	0.7473
	[0,1,2,3,4,5,6,7]	Hyb	0.4229	0.5392	0.6031	0.6590
		Ang	0.4634	0.5415	0.5880	0.6015
		Amp	0.2915	0.3910	0.4181	0.4301
		Re3	**0.5728**	**0.6454**	**0.7069**	0.7288
		Re2	0.4623	0.6141	0.6967	**0.7320**
	[1,2,3,4,5,6,7,8]	Hyb	0.3975	0.5803	0.6143	0.6444
		Ang	0.3158	0.4029	0.4644	0.5234
		Amp	0.2207	0.3170	0.3858	0.4118
		Re3	**0.4791**	**0.6051**	**0.6218**	**0.6727**
		Re2	0.3245	0.5407	0.6080	0.6497
	[2,3,4,5,6,7,8,9]	Hyb	0.3486	0.4356	0.4660	0.5129
		Ang	0.3129	0.4715	0.4970	0.5517
		Amp	0.2426	0.2859	0.3566	0.3837

From Figs. 8 and 9, we can see that for all settings of the number of hidden layers, the *Accuracy* of two repetitive amplitude encodings (i.e. Re3 and Re2) is not only significantly higher than that of other encoding methods on selected datasets, but also shows a obvious upward trend with the increase of the number of hidden layers. In addition, from Table 4, we can see that for all settings of the number of hidden layers, the *F1-score* of two repetitive amplitude encodings (i.e. Re3 and Re2) significantly outperformed the other three encoding methods. In terms of hybrid encoding, angle encoding, and amplitude encoding, it is clear that, hybrid encoding is relatively close to angle encoding, with both being significantly better than amplitude encoding.

**Fig. 10:**
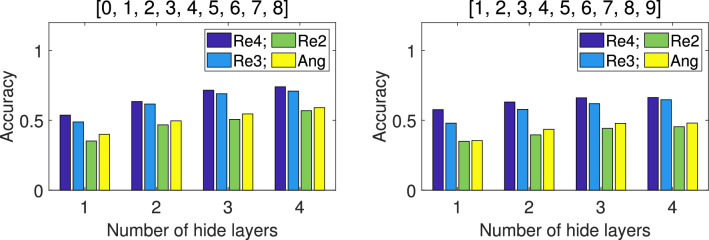
Comparison of *Accuracy* under different encodings for the 9-class test set. The results shown are the mean *Accuracy* of 5 independent QNN training.

**Fig. 11:**
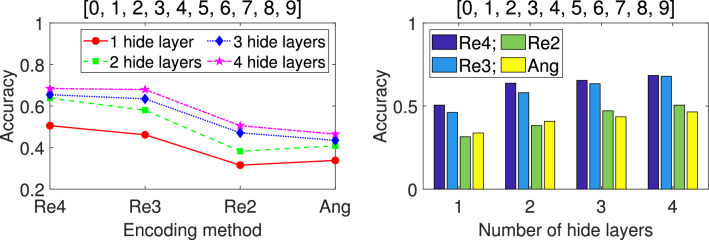
Comparison of *Accuracy* under different encodings for the 10-class test set. The results shown are the mean *Accuracy* of 5 independent QNN training.

For the 9-class classification, we select 2 sample sets based on combinations of different digits, while for 10-class classification, all sample data is used. Obviously, the 9-qubit QNN is no longer suitable, so we use the 12-qubit model shown in Fig. 7 instead. For repetitive amplitude encoding, we divide the 12 qubits into 4 groups $(|\alpha _0\rangle , |\alpha _1\rangle , |\alpha _2\rangle )$, $(|\alpha _3\rangle , |\alpha _4\rangle , |\alpha _5\rangle )$, $(|\alpha _6\rangle , |\alpha _7\rangle , |\alpha _8\rangle )$, and $(|\alpha _9\rangle , |\alpha _{10}\rangle , |\alpha _{11}\rangle )$, which can encode 8 classical data. We can also divide them into 3 groups $(|\alpha _0\rangle , |\alpha _1\rangle , |\alpha _2\rangle , |\alpha _3\rangle )$, $(|\alpha _4\rangle , |\alpha _5\rangle , |\alpha _6\rangle , |\alpha _7\rangle )$, and $(|\alpha _8\rangle , |\alpha _9\rangle , |\alpha _{10}\rangle , |\alpha _{11}\rangle )$, which can encode 16 classical data. We can even divide them into 2 groups $(|\alpha _0\rangle , |\alpha _1\rangle , |\alpha _2\rangle , |\alpha _3\rangle , |\alpha _4\rangle , |\alpha _5\rangle )$ and $(|\alpha _6\rangle , |\alpha _7\rangle , |\alpha _8\rangle , |\alpha _9\rangle , |\alpha _{10}\rangle , |\alpha _{11}\rangle )$, which can encode 64 classical data. According to the previous experimental results, amplitude encoding (Amp) has the worst performance, so in the subsequent result comparison, we did not provide the experimental results of amplitude coding (Amp). Hybrid encoding (Hyb) is relatively close to angle encoding (Ang), so we only use angle encoding for comparison with three repetitive amplitude encoding (i.e. Re4, Re3, and Re2), at this time, it can encode 12 classical data. The comparison schemes are the same as above. The mean *Accuracy* of 5 independent training under different encoding methods for the test set are shown in Fig. 10 and 11, and the mean *F1-score* of 5 independent training under each encoding for the test set are shown in Table 5.

**Table 5:** Comparison of *F1-score* under different encodings for the 9 and 10 classes test set. The results shown are the mean *F1-score* of the test set for 5 independent training. The number with the highest *Accuracy* among the all encoding methods is represented in bold.

Number of class	Test set	Encode	1 hidden layer	2 hidden layers	3 hidden layers	4 hidden layers
9		Re4	**0.4845**	**0.6140**	**0.7012**	**0.7224**
	[0,1,2,3,4,5,6,7,8]	Re3	0.4638	0.5926	0.6690	0.6975
		Re2	0.2902	0.4138	0.4702	0.5356
		Ang	0.3543	0.4702	0.5260	0.5769
		Re4	**0.5579**	**0.6172**	**0.6381**	0.6401
	[1,2,3,4,5,6,7,8,9]	Re3	0.4620	0.5626	0.5937	**0.6409**
		Re2	0.2978	0.3460	0.3842	0.4145
		Ang	0.2910	0.3941	0.4555	0.4347
10		Re4	**0.4499**	**0.6228**	**0.6407**	**0.6777**
	[0,1,2,3,4,5,6,7,8,9]	Re3	0.4336	0.5711	0.6236	0.6715
		Re2	0.2676	0.3192	0.4275	0.4453
		Ang	0.2923	0.3674	0.3955	0.4307

These figures and tables show similar results to those of classification of 7 to 8 classes. The performance of two repetitive amplitude encodings Re4 and Re3 is close, and both are significantly better than angle encoding Ang and repetitive amplitude encoding Re2. It is also worth noting that Ang even outperforms Re2, but there is still a large gap with Re3 and Re4.

#### Statistical analysis of experimental results

In order to fully verify the advantages of repetitive amplitude encoding, this section conducted ANOVA tests on the experimental results of different encoding methods using SPSS software. The test is divided into three groups to examine whether there is a significant difference in the *Accuracy* of classes $2-6$, $7-8$, and $9-10$, respectively.

**Fig. 12:**
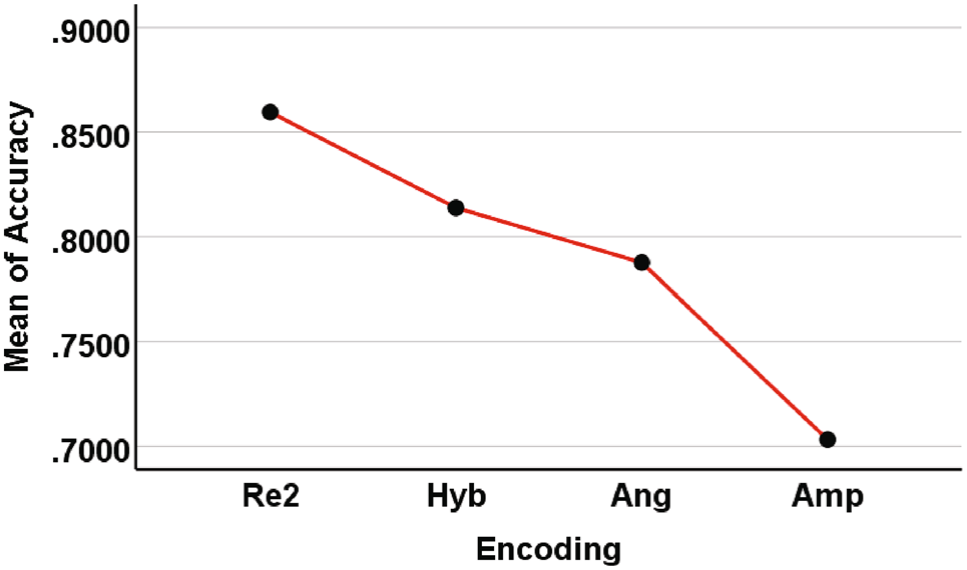
The mean *Accuracy* of four encoding accuracies ($2-6$ classes).

For $2-6$ classes, we used four encoding methods (Re2, Hyb, Ang, Amp). For ease of analysis, we integrated all the results of each encoding method together, including different numbers of classes (2, 3, 4, 5, 6), different datasets ([0, 1], [3, 4], [6, 7], [9, 0], etc.), QNN with different hidden layers (1, 2, 3, 4), and the number of independent runs per experiment (5). Therefore, each encoding method has 400 pieces of *Accuracy* data. The ANOVA test results of the four encoding methods are shown in Tables 6 and 7 and Fig. 12.

**Table 6:** Descriptives of four encoding methods for $2-6$ classes.

Encoding	N	Mean	Std. deviation	Std. error	Lower bound	Upper bound	Minimum	Maximum
Re2	400	0.859572	0.1041931	0.0052097	0.849330	0.869814	0.5661	0.9986
Hyb	400	0.813800	0.1305280	0.0065264	0.800969	0.826630	0.3896	0.9986
Ang	400	0.787600	0.1539254	0.0076963	0.772470	0.802731	0.3854	0.9995
Amp	400	0.703158	0.1664027	0.0083201	0.686801	0.719515	0.3780	0.9915
Total	1600	0.791032	0.1517282	0.0037932	0.783592	0.798473	0.3780	0.9995

**Table 7:** Multiple comparisons results obtained from ANOVA for $2-6$ classes.

(I) Enoding	(J) Encoding	Mean difference (I-J)	Std.error	Sig.	95% confidence interval	
					Lower Bound	Upper Bound
Re2	Hyb	.0457725*	0.0083507	0.000	0.023744	0.067801
	Ang	.0719718*	0.0092937	0.000	0.047450	0.096493
	Amp	.1564143*	0.0098166	0.000	0.130510	0.182319
Hyb	Re2	$-$.0457725*	0.0083507	0.000	$-$0.067801	$-$0.023744
	Ang	.0261992	0.0100909	0.056	$-$0.000418	0.052817
	Amp	.1106418*	0.0105744	0.000	0.082747	0.138537
Ang	Re2	$-$.0719718*	0.0092937	0.000	$-$0.096493	$-$0.047450
	Hyb	$-$.0261992	0.0100909	0.056	$-$0.052817	0.000418
	Amp	.0844425*	0.0113339	0.000	0.054548	0.114337
Amp	Re2	$-$.1564143*	0.0098166	0.000	$-$0.182319	$-$0.130510
	Hyb	$-$.1106418*	0.0105744	0.000	$-$0.138537	$-$0.082747
	Ang	$-$.0844425*	0.0113339	0.000	$-$0.114337	$-$0.054548

*The mean difference is significant at the 0.05 level.

Table 6 presents the results of the descriptive statistics, showing the number of cases involved in the analysis, mean, standard deviation, standard error, $95\%$ confidence interval of mean, minimum value, and maximum value, respectively, according to four different encodings and totals. From Table 6, it can be seen that the mean of the *Accuracy* of repetitive amplitude encoding is the highest and the standard deviation is the smallest. Table 7 shows the results of multiple comparisons. Compare one encoding with the other three encodings in sequence to examine the significance of mean differences. From Table 7, it can be seen that there is a significant difference in the mean *Accuracy* of Re2 compared to Hyb, Ang, and Amp (the significance is much lower than the usual significance *P* value of 0.05, so the null hypothesis of equal means is significantly rejected). The mean of Hyb is significantly different from Re2 and Amp, but not significantly different from Ang (significant *P* value is $0.056 > 0.05$). The difference between Amp and the other three encodings is significant, but the difference values are all negative, making it the worst performing among the four encodings. From the mean *Accuracy* shown in Fig. 12, it can be seen that Re2 is significantly higher, Amp is significantly lower, Hyb and Ang are located in the middle, and the difference between the two is not significant.

**Fig. 13:**
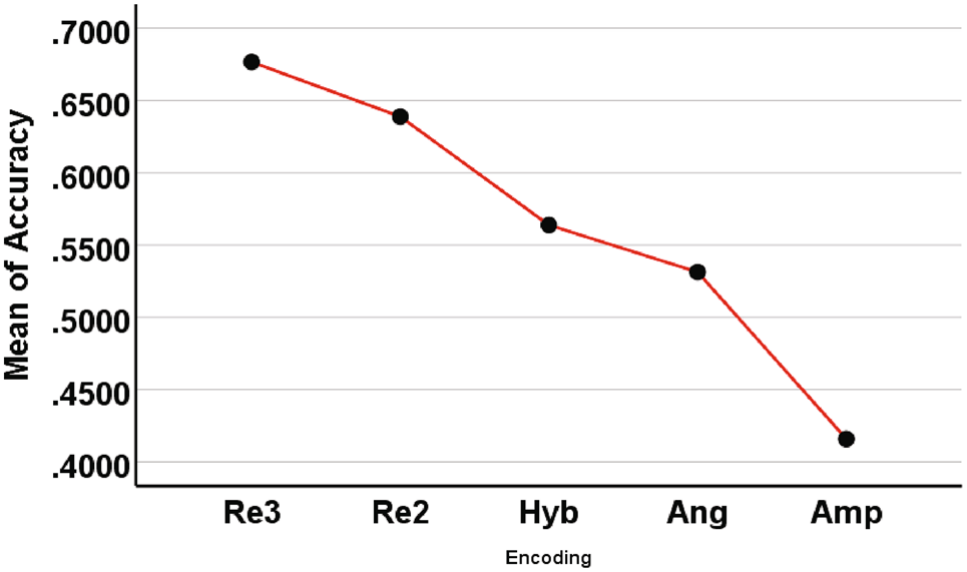
The mean *Accuracy* of five encoding accuracies ($7-8$ classes).

For $7-8$ classes, we used five encoding methods (Re3, Re2, Hyb, Ang, Amp). For ease of analysis, we integrated all the results of each encoding method together, including different numbers of classes (7, 8), different datasets ([1, 2, 3, 4, 5, 6, 7], [2, 3, 4, 5, 6, 7, 8], [3, 4, 5, 6, 7, 8, 9], etc.), QNN with different hidden layers (1, 2, 3, 4), and the number of independent runs per experiment (5). Therefore, each encoding method has 120 pieces of *Accuracy* data. The ANOVA test results of the four encoding methods are shown in Tables 8 and 9 and Fig. 13.

**Table 8:** Descriptives of four encoding methods for $7-8$ classes.

					95% confidence interval			
Encoding	N	Mean	Std. deviation	Std. error	Lower bound	Upper bound	Minimum	Maximum
Re3	120	0.676714	0.0772170	0.0070489	0.662757	0.690672	0.5044	0.8276
Re2	120	0.638938	0.1136545	0.0103752	0.618394	0.659482	0.3451	0.8161
Hyb	120	0.563889	0.0875767	0.0079946	0.548059	0.579719	0.3494	0.7334
Ang	120	0.531402	0.0881105	0.0080434	0.515475	0.547328	0.3499	0.6857
Amp	120	0.415951	0.0587896	0.0053667	0.405324	0.426577	0.2848	0.5346
Total	600	0.565379	0.1256008	0.0051276	0.555309	0.575449	0.2848	0.8276

**Table 9:** Multiple comparisons results obtained from ANOVA for $7-8$ classes.

(I) Enoding	(J) Encoding	Mean difference (I-J)	Std.error	Sig.	95% confidence interval	
					Lower bound	Upper bound
Re3	Re2	.0377758*	0.0125432	0.029	0.002286	0.073266
	Hyb	.1128250*	0.0106584	0.000	0.082702	0.142948
	Ang	.1453125*	0.0106950	0.000	0.115085	0.175540
	Amp	.2607633*	0.0088594	0.000	0.235711	0.285815
Re2	Re3	$-$.0377758*	0.0125432	0.029	$-$0.073266	$-$0.002286
	Hyb	.0750492*	0.0130980	0.000	0.038014	0.112085
	Ang	.1075367*	0.0131278	0.000	0.070418	0.144655
	Amp	.2229875*	0.0116810	0.000	0.189875	0.256100
Hyb	Re3	$-$.1128250*	0.0106584	0.000	$-$0.142948	$-$0.082702
	Re2	$-$.0750492*	0.0130980	0.000	$-$0.112085	$-$0.038014
	Ang	.0324875*	0.0113406	0.045	0.000441	0.064534
	Amp	.1479383*	0.0096289	0.000	0.120692	0.175185
Ang	Re3	$-$.1453125*	0.0106950	0.000	$-$0.175540	$-$0.115085
	Re2	$-$.1075367*	0.0131278	0.000	$-$0.144655	$-$0.070418
	Hyb	$-$.0324875*	0.0113406	0.045	$-$0.064534	$-$0.000441
	Amp	.1154508*	0.0096694	0.000	0.088089	0.142813
Amp	Re3	$-$.2607633*	0.0088594	0.000	$-$0.285815	$-$0.235711
	Re2	$-$.2229875*	0.0116810	0.000	$-$0.256100	$-$0.189875
	Hyb	$-$.1479383*	0.0096289	0.000	$-$0.175185	$-$0.120692
	Ang	$-$.1154508*	0.0096694	0.000	$-$0.142813	$-$0.088089

*The mean difference is significant at the 0.05 level

From Table 8, it can be seen that among the five encoding methods, the two repeated amplitude encodings Re3 and Re2 rank in the top two, followed by Hyb, Ang, Amp. However, Re2 has a slightly larger standard deviation. According to the multiple comparison results given in Table 9, the mean differences between the five encoding methods are significant (significance is lower than the statistical threshold $P=0.05$, significantly rejecting the null hypothesis of equal means). Figure 13 visually displays the mean differences of five encodings.

**Fig. 14:**
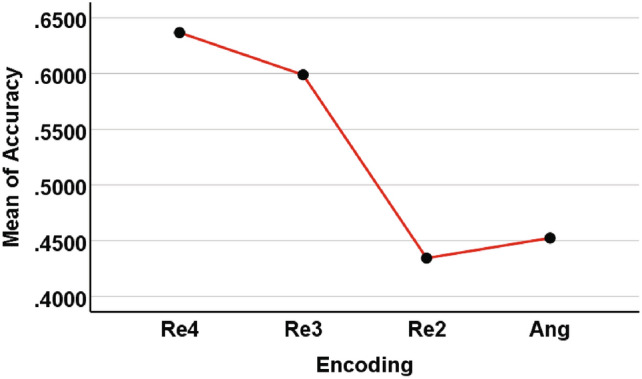
The mean *Accuracy* of four encoding accuracies ($9-10$ classes).

For $9-10$ classes, we used four encoding methods (Re4, Re3, Re2, Ang). For ease of analysis, we integrated all the results of each encoding method together, including different numbers of classes (9, 10), different datasets ([0, 1, 2, 3, 4, 5, 6, 7, 8], [1, 2, 3, 4, 5, 6, 7, 8, 9], [0, 1, 2, 3, 4, 5, 6, 7, 8, 9]), QNN with different hidden layers (1, 2, 3, 4), and the number of independent runs per experiment (5). Therefore, each encoding method has 60 pieces of *Accuracy* data. The ANOVA test results of the four encoding methods are shown in Tables 10 and 11 and Fig. 14.

**Table 10:** Descriptives of four encoding methods for $9-10$ classes.

Encoding	N	Mean	Std. deviation	Std. error	95% confidence interval		Minimum	Maximum
					Lower bound	Upper bound		
Re4	60	0.636817	0.0690119	0.0089094	0.618989	0.654644	0.4727	0.7658
Re3	60	0.598938	0.0824392	0.0106429	0.577642	0.620235	0.4289	0.7221
Re2	60	0.434327	0.0790636	0.0102071	0.413902	0.454751	0.3040	0.5928
Ang	60	0.452357	0.0758443	0.0097915	0.432764	0.471949	0.3152	0.6138
Total	240	0.530610	0.1169833	0.0075512	0.515734	0.545485	0.3040	0.7658

**Table 11:** Multiple comparisons results obtained from ANOVA for $9-10$ classes.

(I) Enoding	(J) Encoding	Mean difference (I-J)	Std.error	Sig.	95% confidence interval	
					Lower bound	Upper bound
Re4	Re3	.0378783*	0.0140126	0.007	0.010273	0.065484
	Re2	.2024900*	0.0140126	0.000	0.174884	0.230096
	Ang	.1844600*	0.0140126	0.000	0.156854	0.212066
Re3	Re4	$-$.0378783*	0.0140126	0.007	$-$0.065484	$-$0.010273
	Re2	.1646117*	0.0140126	0.000	0.137006	0.192217
	Ang	.1465817*	0.0140126	0.000	0.118976	0.174187
Re2	Re4	$-$.2024900*	0.0140126	0.000	$-$0.230096	$-$0.174884
	Re3	$-$.1646117*	0.0140126	0.000	$-$0.192217	$-$0.137006
	Ang	$-$.0180300	0.0140126	0.199	$-$0.045636	0.009576
Ang	Re4	$-$.1844600*	0.0140126	0.000	$-$0.212066	$-$0.156854
	Re3	$-$.1465817*	0.0140126	0.000	$-$0.174187	$-$0.118976
	Re2	.0180300	0.0140126	0.199	$-$0.009576	0.045636

*The mean difference is significant at the 0.05 level.

Table 10 presents descriptive statistics for three types of repetitive amplitude encoding (Re4, Re3, Re2) and angle encoding (Ang). Among them, Re4 and Re3 rank in the top 2, while Re2 is lower than angle encoding This indicates that for complex multi classification of classes $9-10$, the effect of only repeating two qubit blocks during encoding is not ideal. The multiple comparison results shown in Table 11 indicate significant differences between Re4 and the other three encodings, as well as between Re3 and the other three encodings. However, there is no significant difference between Re2 and Ang (*P* value for significance is $0.018 < 0.05$). Fig. 14 visually illustrates the mean differences among the four encodings.

#### Quantitative investigation of the superiority of repetitive amplitude encoding

In the handwritten digits classification experiment, the repetitive amplitude encoding exhibit superior performance compared to other encoding methods. In order to quantitatively examine the advantages of repetitive amplitude encoding, we have defined the following indicators. Let $Acc_{Re4}^{S}$, $Acc_{Re3}^{S}$, $Acc_{Re2}^{S}$, $Acc_{Hyb}^{S}$, $Acc_{Ang}^{S}$, and $Acc_{Amp}^{S}$ represent the *Accuracy* of repetitive amplitude encoding (Re4, Re3, and Re2), hybrid encoding (Hyb), angle encoding (Ang), and amplitude encoding (Amp) on sample set *S*, respectively. In terms of *Accuracy*, the quantization indicator for repetitive amplitude encoding exceeding other encodings are defined as the following.

26 $$\begin{aligned} A_{encod_2}^{encod_1}=\frac{1}{|\Omega |}\sum \limits _{S\in \{\Omega \}}(Acc_{encod_1}^{S}-Acc_{encod_2}^{S}), \end{aligned}$$

where $encod_1\in${Re4, Re3, Re2}, $encod_2\in${Hyb, Ang, Amp}, and

27 $$\begin{aligned} \Omega =\left\{ \begin{array}{ll} \{[0,1],~~[3,4],~~[6,7],~~[9,0]\}, \!\! & \!\! 2-class,\\ \{[0,3,6],~~[1,4,7],~~[2,5,8],~~[3,6,9]\}, \!\! & \!\! 3-class,\\ \{[1,2,3,4],~~[1,3,5,7],~~[2,4,6,8],~~[5,6,7,8]\}, \!\! & \!\! 4-class,\\ \{[1,2,3,4,5],~~[1,3,5,7,9],~~[2,4,6,8,0],~~[6,7,8,9,0]\}, \!\! & \!\! 5-class,\\ \{[1,2,3,4,5,6],~~[2,3,4,5,6,7],~~[3,4,5,6,7,8],~~[4,5,6,7,8,9]\}, \!\! & \!\! 6-class,\\ \{[1,2,3,4,5,6,7],~~[2,3,4,5,6,7,8],~~[3,4,5,6,7,8,9]\}, \!\! & \!\! 7-class,\\ \{[0,1,2,3,4,5,6,7],~~[1,2,3,4,5,6,7,8],~~[2,3,4,5,6,7,8,9]\}, \!\! & \!\! 8-class,\\ \{[0,1,2,3,4,5,6,7,8],~~[1,2,3,4,5,6,7,8,9]\}, \!\! & \!\! 9-class,\\ \{[0,1,2,3,4,5,6,7,8,9]\}, \!\! & \!\! 10-class. \end{array} \right. \end{aligned}$$

Based on the above definition, the quantitative results of repetitive amplitude encoding being superior to other encodings are shown in Table 12.

**Table 12:** The quantitative results of repetitive amplitude encoding being superior to other encodings.

Class	$A_{encod_2}^{encod_1}$	1 hidden layer (%)	2 hidden layers (%)	3 hidden layers (%)	4 hidden layers (%)
2	$A_{Hyb}^{Re2}$	3.310	0.670	0.210	0.490
	$A_{Ang}^{Re2}$	2.280	0.060	0.060	0.430
	$A_{Amp}^{Re2}$	4.760	3.640	3.270	2.880
3	$A_{Hyb}^{Re2}$	5.430	3.960	2.500	1.630
	$A_{Ang}^{Re2}$	5.300	3.150	2.160	2.460
	$A_{Amp}^{Re2}$	12.74	13.01	12.34	11.92
4	$A_{Hyb}^{Re2}$	8.020	6.570	4.190	3.960
	$A_{Ang}^{Re2}$	14.94	10.08	6.280	4.850
	$A_{Amp}^{Re2}$	24.48	21.22	18.93	19.03
5	$A_{Hyb}^{Re2}$	6.360	8.410	5.240	3.310
	$A_{Ang}^{Re2}$	11.75	11.46	8.730	6.530
	$A_{Amp}^{Re2}$	19.15	17.67	18.82	18.62
6	$A_{Hyb}^{Re2}$	11.67	6.440	4.600	4.560
	$A_{Ang}^{Re2}$	17.36	15.05	10.48	10.55
	$A_{Amp}^{Re2}$	21.31	23.11	22.23	23.67
7	$A_{Re2}^{Re3}$	7.440	0.520	-1.780	-1.21
	$A_{Hyb}^{Re3}$	12.65	9.200	8.480	8.510
	$A_{Ang}^{Re3}$	17.12	12.32	10.25	10.56
	$A_{Amp}^{Re3}$	25.98	26.71	25.65	25.89
8	$A_{Re2}^{Re3}$	12.68	7.720	3.250	1.610
	$A_{Hyb}^{Re3}$	14.96	13.63	11.67	11.16
	$A_{Ang}^{Re3}$	15.59	18.53	16.43	15.45
	$A_{Amp}^{Re3}$	22.80	26.99	27.05	27.54
9	$A_{Re3}^{Re4}$	7.260	3.520	3.320	2.310
	$A_{Re2}^{Re4}$	20.56	20.15	21.37	19.03
	$A_{Ang}^{Re4}$	17.95	16.67	17.67	16.60
10	$A_{Re3}^{Re4}$	4.370	5.770	2.000	0.500
	$A_{Re2}^{Re4}$	19.01	25.54	18.35	17.87
	$A_{Ang}^{Re4}$	16.72	22.95	21.98	21.94

Firstly, in Table 12, the difference in *accuracy* between repetitive amplitude encoding and all other encodings (i.e. $A_{Hyb}^{Re2}$, $A_{Ang}^{Re2}$, $A_{Amp}^{Re2}$, $A_{Hyb}^{Re3}$, $A_{Ang}^{Re3}$, $A_{Amp}^{Re3}$, $A_{Ang}^{Re4}$) is always positive, indicating that the mean *Accuracy* of repetitive amplitude encoding is higher than other encoding methods.

Secondly, from a horizontal perspective of the table, as the number of hidden layers increases (i.e. the depth of parameterized quantum circuits increases), the vast majority of values show a decreasing trend. This indicates that as the number of layers increases, the gap between various encoding methods tends to decrease.

Thirdly, from the vertical perspective of the table, as the number of classes increases (i.e., the width of the parameterized quantum circuit increases), most of the values in the table generally tend to increase, although some values occasionally decrease. This indicates that the more classes there are, the more obvious the advantages of repetitive amplitude encoding compared to other encodings.

Fourthly, for repetitive amplitude encoding, the more repetitions (i.e. the more number of blocks of qubits) the better the performance, but this difference is not significant when the number of classes is small. For example, for 7-class classification and 8-class classification, the vast majority of $A_{Re2}^{Re3}$ are positive, but their absolute value is small, and occasionally negative values appear. However, as the number of classes increases (i.e. the width of parameterized quantum circuits continues to increase), the gap between different repetitive amplitude encodings will increase. For example, for 9-class classification and 10-class classification, all $A_{Re3}^{Re4}$ are positive and their absolute values are very small, but the values of all $A_{Re2}^{Re4}$ become very large. This shows that repetitive amplitude encoding that relies solely on the superposition of two blocks of qubits (i.e. Re2) no longer meets the requirements of multi-class classification.

Finally, in general, amplitude encoding has the worst performance because it does not introduce any nonlinear mapping to the encoded data. The performance of angle encoding is close to that of hybrid encoding. Especially for multi-class classification (such as 10-class classification), angle encoding even exceeds the repetition amplitude encoding Re2 based solely on the superposition of two blocks of qubits. Therefore. for multi-class classification, in addition to repetitive amplitude encoding, angle encoding is also a potential alternative solution.

### Comparison with the recent state-of-the-art quantum neural network models

To further validate the classification capability of our proposed model, we selected three state-of-the-art quantum neural network classification models from recent literature for comparative analysis.

The first model is the “novel modular Quantum Neural Network (mQNN)” proposed by Yan et al., which was specifically designed for binary classification tasks on the MNIST dataset. Using the classification of handwritten digits 0 and 1 as an example, this model demonstrates the significant potential of quantum computing in image classification tasks^45^.

The second model is the “Quantum Convolutional Neural Network with Interaction Layer (QCNNIL)”, which significantly enhances classification performance by introducing a novel interaction layer utilizing three-qubit interactions between convolutional layers. Experimental results on both binary (0,1) and ternary (0,1,2) classification tasks from the MNIST dataset surpassed the existing state-of-the-art approaches^46^.

The third model is the “Scalable Quantum Convolutional Neural Network (SQCNN)”, which incorporates classical CNN principles while better learning features through superposition and entanglement between quantum gates. Specifically, the SQCNN system enables multiple independent quantum devices to perform parallel feature extraction. The model’s advantages were verified through three binary classification tasks ((0,1), (2,5), and (3,6)) on the MNIST dataset^47^.

The performance comparison of repetitive amplitude encoding-based quantum neural networks (RAEQNN) with mQNN, QCNNIL, and SQCNN is shown in Tables 13, 14 and 15, respectively. In these three tables, the results of the comparison models are all derived from the corresponding references.

**Table 13:** Performance comparison of RAEQNN and mQNN on handwritten digit dataset (0,1). The results shown are the mean *Accuracy* of the test set for 5 independent training. The number with the highest *Accuracy* among the all encoding methods is represented in bold.

RAEQNN					mQNN				
Layers	Qubits	Parameters	Epoch	Accuracy	Layers	Qubits	Parameters	Epoch	Accuracy
1	6	16	100	**0.9971**	4	10	4	200	0.9305
2	6	26	100	**0.9969**	4	18	16	200	0.9665
3	6	36	100	**0.9965**	4	22	64	200	0.9800
4	6	46	100	**0.9975**	–	–	–	–	–

**Table 14:** Performance comparison of RAEQNN and QCNNIL on handwritten digit dataset (0, 1) and (0, 1, 2). The results shown are the mean *Accuracy* of the test set for 5 independent training. The number with the highest *Accuracy* among the all encoding methods is represented in bold.

Dataset	RAEQNN					QCNNIL				
	Layers	Qubits	Parameters	Epoch	Accuracy	Layers	Qubits	Parameters	Epoch	Accuracy
(0, 1)	4	6	46	100	**0.9975**	7	10	50	1000	0.9900
(0, 1, 2)	4	6	46	100	**0.9565**	7	18	53	1000	0.9005

**Table 15:** Performance comparison of RAEQNN and SQCNN on handwritten digit dataset (0, 1), (2, 5) and (3, 6). The results shown are the mean *Accuracy* of the test set for 5 independent training. The number with the highest *Accuracy* among the all encoding methods is represented in bold.

Model	Qubits	(0, 1)			(2, 5)			(3, 6)		
		Accuracy	F1-score	Recall	Accuracy	F1-score	Recall	Accuracy	F1-score	Recall
RAEQNN	6	**0.9975**	**0.9975**	**0.9975**	**0.9813**	**0.9813**	**0.9812**	**0.9876**	**0.9876**	**0.9876**
SQCNN	64	0.9968	0.9966	0.9947	0.9650	0.9673	0.9632	0.9752	0.9757	0.9752

The comparative results demonstrate that RAEQNN not only has a simpler model structure (with fewer qubits and fewer trainable parameters), but also achieves the best classification performance. The results demonstrate that our proposed RAEQNN outperforms existing quantum techniques for image classification.

### Experimental results of reservoir lithology identification

**Fig. 15:**
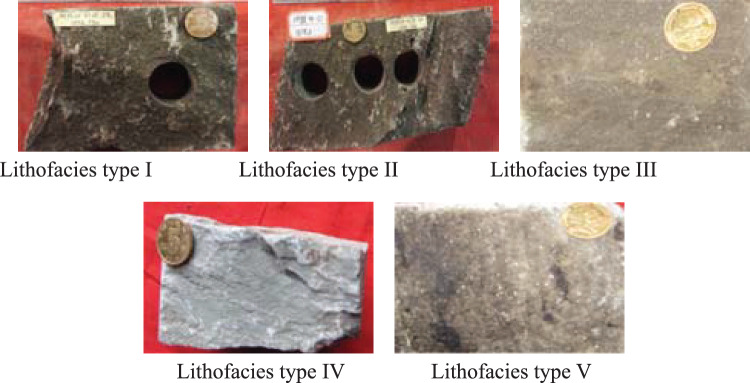
6 types of lithofacies obtained from core analysis results.

In this experiment, we take a block of Daqing Oilfield as the target area. Based on the core data of 17 coring wells, the following 5 main developed lithofacies are determined from the two aspects of development degree and oil-bearing properties (as shown in Fig. 15): (I) trough-shaped cross-bedded fine sandstone; (II) plate-like cross-bedded fine sandstone; (III) parallel/oblique bedding fine sandstone; (IV) wavy bedding fine sandstone; (V) horizontal bedding siltstone.

For each lithofacies, we directly use the logging responses in its depth range to construct the training samples. We selected 15 coring wells to train the repetitive amplitude encoding-based QNN and establish the mapping relationship between logging responses and lithofacies types, and the remaining 2 coring wells were used to test the lithofacies identification ability of QNN. To ensure sample balance, for each type of lithology, 300 reservoir samples were randomly selected from 15 training wells to form the training set, and then 100 reservoir samples were randomly selected from 2 testing wells to form the testing set. Therefore, the number of training samples and testing samples are 1500 and 500, respectively.

#### Construction of image samples

**Fig. 16:**
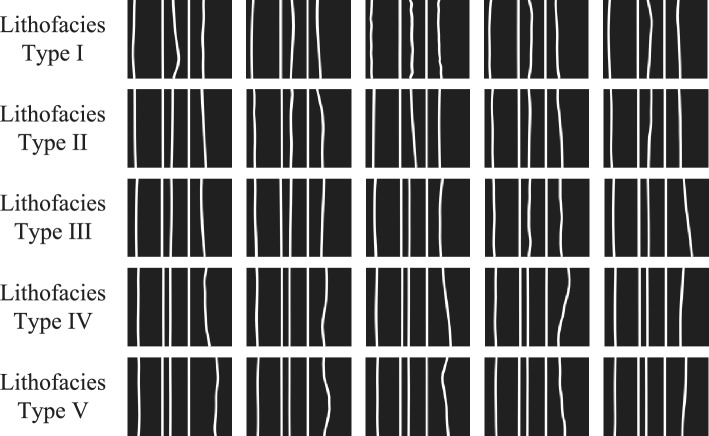
25 image samples randomly selected from five types of lithofacies.

Based on knowledge in the field of reservoir identification, the logging responses closely related to the lithofacies type mainly include natural gamma (GR), deep investigate lateral resistivity (LLD), acoustic time difference (AC), spontaneous potential (SP), borehole diameter (CAL)^48^. This paper also uses these five indicators to construct sample data. First, according to the analysis results of core data, the types of various lithofacies and the corresponding depth range are determined. At this time, the lithofacies type is the expected output sample (i.e. label). Then, the input samples are constructed by using the logging response within each lithofacies depth range. For image samples, we first plot the logging responses in its depth range into curves images, and then scale them to a uniform size $128\times 128$. Some image samples are shown in Fig. 16. These image samples can be directly submitted to the repetitive amplitude encoding-based QNN.

#### Training and testing

**Fig. 17:**
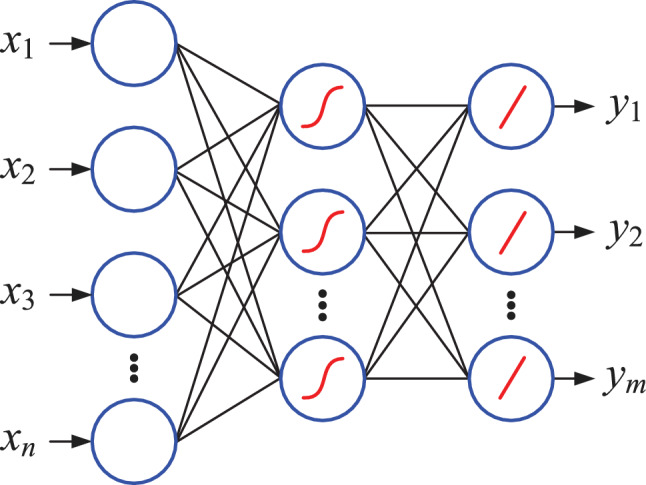
Multi-layer perceptron model.

In this experiment, there are five types of lithology in the reservoir samples, so a 6-qubit QNN as shown in Fig. 3 can be used. To support the potential advantages of repetitive amplitude encoding-based QNN in classification, we present the experimental results of classical Multi-Layer Perceptron (MLP) in this section. The general model of three-layer MLP is shown in Fig. 17, where the number of input layer nodes *n* is determined by the number of input sample features, the number of hidden layer nodes is generally determined by multiple independent experiments, and the number of output layer nodes *m* is equal to the number of classes in the dataset. The hidden layer and output layer use *Sigmoid* and *linear* function as excitation functions, respectively.

**Fig. 18:**
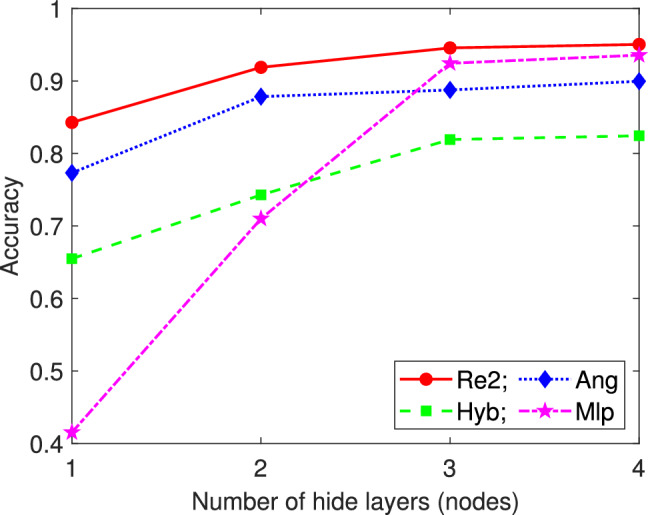
Comparison of *Accuracy* under different encodings for test set. The results shown are the mean *Accuracy* of 5 independent QNN training.

To ensure fair comparison, an autoencoder is used to reduce the dimensionality of all MLP sample images to an 8-dimensional vector, and so, the MLP has 8 input nodes. The number of hide nodes is set to 1, 2, 3, and 4, respectively. To fully evaluate the excellent performance of repetitive amplitude encoding, we also present experimental results of hybrid encoding and angle encoding. For repetitive amplitude encoding, hybrid encoding, and angle encoding, autoencoders are also used to implement dimensionality reduction, and the scheme is the same as the MNIST experiment. The number of hidden layers in QNN is set to 1, 2, 3, and 4, respectively. Both QNN and MLP employ Mini-Batch Gradient Descent training, where the batch-size is set to 50. QNN and MLP employ “nesterov” and “adam” optimizers to optimize model parameters respectively. The training epochs are all set to 100. The learning rate is set to $10^{-2}$, and the loss function adopts *cross* *entropy*. In order to reduce the randomness of the experimental results, 5 training were conducted independently under each parameter setting, and the average value of the 5 evaluation results was taken for comparison. The mean *Accuracy* of 5 independent evaluations for the test set are shown in Fig. 18, and the same results are also shown in Tab. 16. The performance comparison between QNN and MLP is only meaningful if the two models have the same (or similar) number of parameters. Therefore, to enhance the fairness of the comparison, we also count the number of parameters under different hidden layer settings for the two models, and show them in square brackets after the numbers in the Table 16.

**Table 16:** Comparison of *Accuracy* between MLP and QNN with Different Encoding on the Test Set. The results shown are the mean *Accuracy* of the test set for 5 independent training. The number with the highest *Accuracy* among the all encoding methods is represented in bold. The number in square brackets after the number represents the number of QNN and MLP model parameters.

Model	Encode	1 hidden layer (node)	2 hidden layers (nodes)	3 hidden layers (nodes)	4 hidden layers (nodes)
QNN	Re2	**0.8427** [16]	**0.9188** [26]	**0.9456** [36]	**0.9504** [46]
	Hyb	0.6548 [16]	0.7427 [26]	0.8192 [36]	0.8244 [46]
	Ang	0.7732 [16]	0.8784 [26]	0.8876 [36]	0.8996 [46]
MLP	—	0.4152 [19]	0.7100 [33]	0.9244 [47]	0.9356 [61]

From Fig. 18, when the model parameters are small ($1-2$ hidden layers), repetitive amplitude encoding not only significantly outperforms hybrid encoding and angle encoding, but also significantly outperforms MLP. When there are many model parameters ($3-4$ hidden layers), although MLP surpasses hybrid encoding and angle encoding, it is still inferior to repetitive amplitude encoding. Table 16 not only shows the same comparison results, but also the number of parameters in repeatitive amplitude encoding-based QNN is less than that in MLP.

**Fig. 19:**
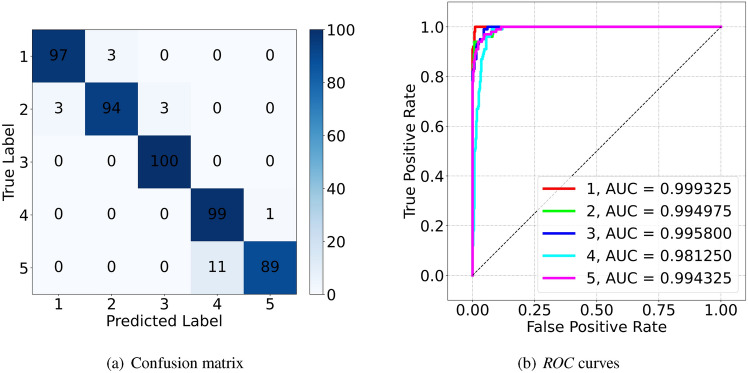
Confusion matrix and *ROC* curves of repetitive amplitude encoding-based QNN with 4 hidden layers.

For repetitive amplitude encoding, taking QNN with 4 hidden layers as an example, the confusion matrix and *ROC* curves for the test set obtained from the third independent training are shown in Fig. 19.

From Fig. 19a, it can be seen that the *Accuracy* of the repeated amplitude encoding on the test set is equal to $\frac{97+94+100+99+89}{500}$ $=95.8\%$, Except for the slightly lower discrimination between classes 4 and 5, the discrimination between other classes is relatively ideal. From Fig. 19b, it can be seen that the *ROC* curves of all 5 classes are far from the diagonal and close to the upper left corner, resulting in an area (*AUC* value) between them and the horizontal axis close to 1. All of these reveal that repetitive amplitude encoding-QNN has great potential in classification tasks.

### Experimental results of IRIS and WINE datasets

The previous experiment investigated the advantages of repetitive amplitude encoding in quantum neural networks for image classification. For image samples, dimensionality reduction is first required. In fact, this method is universal and can be applied to any other classification or recognition problem. In this section, we examined the ability of quantum neural networks based on repetitive amplitude encoding to directly handle low dimensional classification tasks, and compared the classification results with the classic Multi-Layer Perceptron (MLP) network.

#### Classification of IRIS plant

This is perhaps the best known database to be found in the pattern recognition literature. The dataset contains 3 classes of 50 instances each, where each class refers to a type of iris plant (i.e. Iris Setosa, Iris Versicolour, Iris Virginica). One class is linearly separable from the other 2; the latter are NOT linearly separable from each other. Each instance includes four attributes: sepal length; sepal width; petal length; petal width.

Due to the four features of each IRIS sample, they can be encoded as amplitudes of two qubits. To improve the nonlinear mapping ability of the classification model, we use repetitive amplitude encoding (Re3) and classify it using the 6-qubit QNN shown in Fig. 3. The relevant parameter settings are as follows. The number of hidden layers of QNN is set to $kQ=1, 2, 3, 4$, and “nesterov” is employed as the optimizer. MLP adopts a three-layer structure as shown in Fig. 17, with 4 nodes in the input layer and 3 nodes in the output layer, and employs “adam” optimizers to optimize model parameters. To compare the classification performance under different parameter quantities, the number of hidden layer nodes in MLP is set to $kM=1, 2, 3, 4$ respectively. The iteration steps of QNN and MLP are both set to 50, and the learning rates are set to $LR=0.005, 0.01, 0.05$, respectively, and for each learning rate, 20 independent experiments are run on the training set, and then the mean *Accuracy* on the test set is compared. The training/test sample ratio is set to 3 : 2. The comparison of the *Accuracy* of the two models on the test set is shown in Table 17. The bold values in Table 17 indicate that the value is higher than the value compared with it.

**Table 17:** The *Accuracy* for the IRIS test set (average of 20 experiments).

*kQ* or *kM*	Model	Parameter quantity	Mean *Accuracy*		
			$LR=0.005$	$LR=0.01$	$LR=0.05$
1	QNN	16	**0.982**	**0.983**	**0.982**
	MLP	11	0.603	0.686	0.932
2	QNN	26	**0.97.9**	**0.980**	**0.979**
	MLP	19	0.778	0.793	0.943
3	QNN	36	**0.97.8**	**0.978**	**0.977**
	MLP	27	0.805	0.863	0.956
4	QNN	46	**0.97.9**	**0.982**	**0.977**
	MLP	35	0.816	0.904	0.964

The experimental results show that not only is the performance of QNN minimally affected by the number of parameters and learning rate, but the mean *Accuracy* is significantly higher than that of MLP. Especially when QNN has only one hidden layer (with 16 adjustable parameters), the mean *Accuracy* at all three learning rates is above $98\%$, while for MLP, the performance is greatly affected by the learning rate and number of parameters, the highest mean *Accuracy* is only $96.4\%$ (with 35 adjustable parameters).

Next, we use the independent sample T-test to perform statistical analysis on the experimental results. According to the specific settings of the number of hidden layers (nodes), learning rate, and number of independent runs in the experiment, each model generated 240 result data. The results of the independent sample T-test performed on these data are shown in Table 18. According to the results shown in Table 18, the two tailed significance P-values of the mean homogeneity T-test are both less than 0.05 under the assumption of equal variance and non assumption of equal variance, significantly rejecting the null hypothesis of equal mean values. This means that the mean *Accuracy* of the two models is significantly different. These results indicate that for low dimensional small sample dataset with only 150 instances, compared with MLP, QNN based on repetitive amplitude encoding also has significant advantages.

**Table 18:** Independent sample T-test of experimental results of QNN and MLP on IRIS dataset.

				Mean	Std. error	95% confidence interval	
Equal Variances	t	df	Sig. (2-tailed)	Difference	Difference	Lower bound	Upper bound
Assumed	13.987	478	0.000	0.1433767	0.0102506	0.1232348	0.1635185
Not Assumed	13.987	240.449	0.000	0.1433767	0.0102506	0.1231842	0.1635691

#### Classification of WINE dataset

The WINE dataset is a well-known dataset in the field of machine learning and statistics, often used for classification tasks. It contains the results of a chemical analysis of WINE grown in the same region in Italy but derive from three different cultivars. The dataset includes 178 instances, each described by 13 numerical attributes, which represent various chemical properties of the WINE, such as alcohol content, malic acid, ash, and more. The goal is to use these attributes to classify the WINE samples into one of the three cultivars. The WINE dataset is frequently used for testing machine learning algorithms and techniques due to its relatively small size and the clear distinction between classes.

**Fig. 20:**
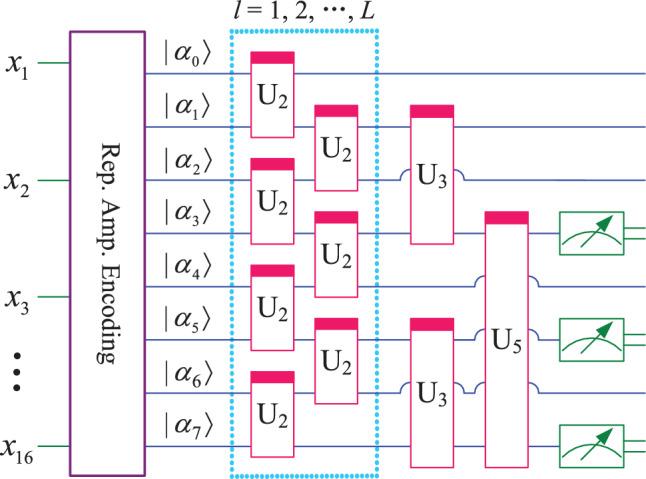
QNN model for classification of the WINE dataset.

To meet the input requirements of QNN, the 13 dimensional data is first interpolated into 16 dimensions using cubic interpolation. The interpolated data can be encoded into probability amplitudes of 16 basis states generated by 4 qubits. Then, classification is implemented using the 8-qubit QNN based on repetitive amplitude encoding (Re2) shown in Fig. 20.

In Fig. 20, the mapping relationship of parameterized quantum circuits in QNN can be written as

28 $$\begin{aligned} map = (I^{\otimes 3}U_5)(I\otimes U_3)^{\otimes 2}((I\otimes U_2^{\otimes 3}\otimes I)(U_2^{\otimes 4}))^l. \end{aligned}$$

The number of samples for the three classes is 59, 71,and 48, with 40, 50, and 30 randomly selected as training sets, and the remaining 19, 21, and 18 as testing sets. The number of hidden layers in QNN is set to $kQ=1, 2, 3, 4$. The structure of MLP is $16-kM-3$, and the number of hidden layer nodes is also set to $kM=1, 2, 3, 4$. The learning rates of the two models are $LR=0.005, 0.01, 0.05$, and the iteration steps are limited to 100. The number of independent experimental rounds for the two models is set to 20, and they each use the same optimizer as before. The mean *Accuracy* of 20 independent experiments is shown in Table 19, in which the bold values indicate that the value is higher than the value compared with it.

**Table 19:** The *Accuracy* for the WINE test set (average of 20 experiments).

*kQ* or *kM*	Model	Parameter quantity	Mean *Accuracy*		
			$LR=0.005$	$LR=0.01$	$LR=0.05$
1	QNN	20	**0.901**	**0.896**	**0.891**
	MLP	23	0.621	0.656	0.768
2	QNN	34	**0.922**	**0.944**	**0.942**
	MLP	43	0.796	0.863	0.915
3	QNN	48	**0.951**	**0.950**	**0.962**
	MLP	63	0.844	0.922	0.941
4	QNN	62	**0.964**	**0.966**	**0.973**
	MLP	83	0.912	0.934	0.952

The experimental results in Table 19 show that even though QNN has fewer parameters than MLP, its mean *Accuracy* is still significantly higher than MLP, especially when the learning rate and number of parameters of the two models are relatively small, this difference is more obvious. The independent sample T-test of the results obtained from the two models is shown in Table 20. The results indicate that the two tailed significance level of the mean equivalence T-test is also less than 0.05, significantly rejecting the null hypothesis of mean equivalence, thus verifying the good adaptability of QNN based on repetitive amplitude encoding to low dimensional classification tasks.

**Table 20:** Independent sample T-test of experimental results of QNN and MLP on WINE dataset.

				Mean	Std. error	95% confidence interval	
Equal Variances	t	df	Sig. (2-tailed)	Difference	Difference	Lower bound	Upper bound
Assumed	10.330	478	0.000	0.0888033	0.0085967	0.0719114	0.1056953
Not Assumed	10.330	276.186	0.000	0.0888033	0.0085967	0.0718800	0.1057267

## Discussion

In the previous section, we presented the experimental results of the proposed method on four datasets in detail. Next, we discuss the reasons behind some phenomena observed in the experiments.

Firstly, when the number of classes in the dataset is fixed, the repetitive amplitude encoding exceeds hybrid encoding, angle encoding, and amplitude encoding. In QNN using parameterized quantum circuits as the basic structure, due to the fact that all quantum gates can only perform linear transformations and only the measurement operation at the output end has nonlinearity, its nonlinear mapping ability is not ideal. In terms of the four types of encoding, amplitude encoding only normalizes the original data without introducing any nonlinearity, so the performance of QNN based on amplitude encoding is the worst. Angle encoding first transforms classical data into angles, and then uses trigonometric functions to convert each classical data into the probability amplitude of a single qubit basis state. The introduction of nonlinearity in this encoding method mainly relies on trigonometric functions. For multiple qubits, the abnormally complex frequency characteristics presented by the product of multiple trigonometric functions disrupt the feature expression in the original data, thereby also affecting the improvement of QNN’s nonlinear mapping ability. Although hybrid encoding introduces nonlinearity through the superposition and entanglement of blocks of qubits, the fact that the classical data encoding the probability amplitudes of the blocks of qubits have different normalization constant directly reduces the fidelity of the original data representation. The repetitive amplitude encoding also uses multiple blocks of qubits and introduces nonlinear mapping through the tensor product of the encoding results of multiple blocks of qubits. However, unlike hybrid encoding, each blocks of qubits uses the same set of classical data, according to Theorem 1, the repetitive amplitude encoding is the most faithful representation of the original data. Hence, it directly avoiding the shortcomings of hybrid encoding. This is the fundamental reason for the excellent performance of QNN based on repetitive amplitude encoding.

Secondly, when the number of hidden layers is fixed, as the number of classes increases, QNN with repetitive amplitude encoding not only consistently outperforms QNN with other encodings, but also this advantage becomes increasingly apparent. The fixed number of layers in QNN is equivalent to the fixed depth of parameterized quantum circuits, while the increase in the number of classes is equivalent to the expansion of the width of parameterized quantum circuits. The increase in the number of classes means an increase in classification difficulty, which provides a suitable scenario for highlighting the advantages of repetitive amplitude encoding. The expansion of the width of parameterized quantum circuits will inevitably lead to an increase in the probability of a barren plateau in the loss function. However, in repetitive amplitude encoding, when the number of qubits in the parameterized quantum circuit increases from *q* to $q+1$, the corresponding range of class numbers that can be increased is $1\sim (2^{q+1}-2^{q})$. In other words, if the increase in the number of classes is less than $(2^{q+1}-2^{q})$, the width of the parameterized quantum circuit remains unchanged. This results in the speed of parameterized quantum circuit width expansion lagging far behind the speed of class increase. So the advantage of repetitive amplitude encoding can to some extent offset the impact of the barren plateau caused by the increase in width of parameterized quantum circuits. Thus, the performance of QNN based on repetitive amplitude encoding becomes increasingly prominent as the number of classes increases.

Thirdly, compared to the widely used angle encoding, the requirement for data dimensionality in repetitive amplitude encoding is much more relaxed. This is because for *n*-dimensional classical data, the required number of qubits for angle encoding is *O*(*n*), while the number of qubits required for repetitive amplitude encoding is only $(\log _2(n))$. Therefore, for current noisy intermediate-scale quantum devices, angle encoding is no longer applicable for high-dimensional classical data (such as 30 dimensions or more). For example, for 32 dimensional classical data, angle encoding requires 32 bits, while using repetitive amplitude encoding Re2 only requires $2\times \log _2(32)=10$ qubits. This indicates that repetitive amplitude encoding has a wider range of applications than angle encoding.

Fourthly, a noteworthy issue is that for repetitive amplitude encoding, although it is possible to introduce nonlinear transform of classical data through the superposition and entanglement of multiple qubit blocks, the number of classical data features that it can encode (i.e. sample dimension) must be an integer power of 2. This limits the universality of this method in practical applications to a certain extent. Assuming that each block in the repetitive amplitude encoding has *d* qubits, it can encode $2^d$ classical data. The solution given in this paper is: if the dimension of the classical data is greater than $2^d$, the dimension can be reduced to $2^d$ by using the dimensionality reduction method. Otherwise, the dimension can be expanded to $2^d$ by using the interpolation method, or by directly adding 0 at the end.

Fifthly, regarding the information capacity of the encoding method. The information capacity of repetitive amplitude encoding is smaller than that of hybrid encoding but larger than that of angle encoding. Nevertheless, our experimental results show that repetitive amplitude encoding has excellent classification performance. The reasons for this, as mentioned earlier, are: first, the encoding itself introduces nonlinearity; second, it eliminates the potential impact of inconsistent normalization constants on classification performance. As for dimensionality reduction of high-dimensional original data, it is not uniquely required by the repetitive amplitude encoding method but is necessary for various quantum encoding methods—primarily to reduce the number of qubits in the quantum system. For low-dimensional data (e.g., the IRIS dataset), dimensionality reduction is entirely unnecessary. Additionally, the nonlinear mapping capability of the network is not generated through preprocessing but rather emerges from the superposition and entanglement between qubits of different blocks.

Sixthly, regarding the potential scalability issues when implementing repetitive amplitude encoding in larger quantum systems, we conducted the following tests. Taking the MNIST binary classification dataset (digits 0 and 1) as an example, we first reduced the original $28\times 28$ amplitude images to 16 dimensions using an autoencoder, which could then be encoded with 4 qubits. Next, we applied repetitive amplitude encoding to these 16 classical data points, with the repetition counts set to 2, 3, 4, 5, and 6, respectively. This required total qubit counts of 8, 12, 16, 20, and 24. By evaluating the accuracy on the test set (980 samples of 0 and 1135 samples of 1), we assessed the scalability of repeated amplitude encoding in larger quantum systems. To simplify the model structure, each QNN used only one hidden layer, with parameter counts of 20, 28, 36, 44, and 52, respectively. To improve experimental efficiency, the number of iterations was set to just 10. Each QNN was independently run 5 rounds, and the average accuracy on the test set was taken as the final result. The average prediction results for the five QNNs with different quantum circuit sizes were 0.9892, 0.9801, 0.9896, 0.9780, and 0.8701, respectively. This reveals that in larger quantum systems, as the number of qubits increases, accuracy tends to saturate or even decline. This phenomenon may be related to the barren plateau problem in QNNs and the more pronounced quantum circuit noise in larger-scale quantum systems. Effectively addressing this issue will help overcome challenges in implementing QNNs on real quantum devices, such as quantum gate errors, decoherence, and their impact on encoding method performance. This is also one of the key research questions we plan to focus on in our next steps.

Finally, this paper only examines the application effect of QNN on classification. Regarding regression, our preliminary attempts indicate that even with the repetitive amplitude encoding strategy proposed in this paper, the effectiveness of QNN in solving regression is still not ideal. The reason for this may be that regression problems require models with stronger nonlinear mapping capabilities to solve. Although the repetitive amplitude encoding strategy introduces a certain degree of nonlinearity to QNN, these nonlinearities are not sufficient to solve complex regression tasks.

## Conclusion

This paper mainly studies a classical data encoding method that can enhance the nonlinear mapping ability of quantum neural networks, a quantum neural network construction method based on 2-qubit ansatzs, and their applications in multi-class classification. The entire QNN are implemented using the “qml.qnode” simulator provided by PennyLane. Model optimization is also performed on a classical computer with the help of quantum operations written in the Python package provided by PennyLane. The important contribution of this paper is the proposal of a repetitive amplitude encoding method for classical data. This method encodes some qubit blocks using the same set of classical data, and effectively introduces nonlinear mapping capability to QNN by utilizing the superposition and entanglement of these qubit blocks. At the same time, due to the fact that the classical data encoding each qubit block is the same, the problem of inconsistent normalization constant of the classical data encoding each qubit block in the hybrid encoding method is perfectly solved. This method has good adaptability to classification (whether binary or multi-classification). Through classification experiments on MNIST datasets, the performance of the proposed method is examined in detail, and the advantages of this method compared with other encoding methods are verified. The experimental results of applying this method to reservoir lithology identification in the field of oil and gas exploration show that compared with classical methods, this method not only improves identification accuracy, but also has fewer control parameters. The application results on two low dimensional classification datasets show that this method has good universality. How to further enhance the nonlinear mapping capability of QNN in order to effectively solve regression tasks is the focus of our next research.

## Supplementary Information

Supplementary Information.

## Data Availability

The datasets used and/or analyzed during the current study is available from the corresponding author on reasonable request.
