# Supplementary material for: A repetitive amplitude encoding method for enhancing the mapping ability of quantum neural networks
Source: Sci Rep. 2025 Sep 1;15:32111. doi: 10.1038/s41598-025-17651-5 (PMC12402107; doi:10.1038/s41598-025-17651-5)
Supplement: Supplementary file 1 — Supplementary Information. [file 41598_2025_17651_MOESM1_ESM.pdf]

Supplementary Material for: [A Repetitive Amplitude Encoding Method for Enhancing the Mapping Ability of Quantum Neural Networks]

Appendix A Parameter tuning for binary classification models

The binary classification model is shown in Supplementary Fig. S1. To simplify the presentation, we derive the parameter update rule based on a single training instance.

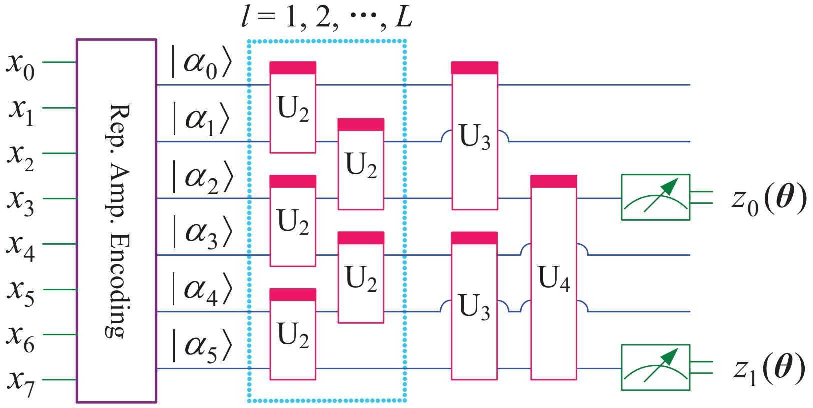

Supplementary Figure S1. QNN model for binary classification.

Let the classical training data be denoted as $\boldsymbol{X} = [x_0, x_1, \cdots, x_7]$. After applying repetitive amplitude encoding, the state of the quantum system is

$$|X\rangle = \left(\sum_{i=0}^{7} x_i |i\rangle\right) \otimes \left(\sum_{j=0}^{7} x_j |j\rangle\right) = \sum_{i=0}^{7}\sum_{j=0}^{7} x_i x_j |ij\rangle = \left[x_0x_0, x_0x_1, \cdots, x_0x_7, \cdots, x_7x_0, x_7x_1, \cdots, x_7x_7\right]^{\mathrm{T}}. \tag{S1}$$

Following Eq. (16) in the main text, the transformations implemented by the quantum circuits in each layer of the model can be comprehensively expressed as $U(\boldsymbol{\theta}) = (I^{\otimes 2} \otimes U_4)U_3^{\otimes 2}((I \otimes U_2^{\otimes 2} \otimes I)U_2^{\otimes 3})^l$, where $\boldsymbol{\theta} = \left[\theta_1, \theta_2, \cdots, \theta_{10l+6}\right]$ represents the model parameters. After the action of $U(\boldsymbol{\theta})$, the state of the quantum system is $|Y\rangle = U|X\rangle = |\bar{\alpha}_0\bar{\alpha}_1\bar{\alpha}_2\bar{\alpha}_3\bar{\alpha}_4\bar{\alpha}_5\rangle = \left[y_0(\boldsymbol{\theta}), y_1(\boldsymbol{\theta}), \cdots, y_{63}(\boldsymbol{\theta})\right]^{\mathrm{T}}$.

According to the principles of quantum computing, the measurement operators on the 2nd and 5th qubits can be written as $\hat{Z}_2 = I^{\otimes 2} \otimes Z \otimes I^{\otimes 3}$ and $\hat{Z}_5 = I^{\otimes 5} \otimes Z$, respectively. The measurement operations and results for these two qubits are

$$\langle \hat{Z}_2 \rangle = \langle Y|\hat{Z}_2|Y\rangle = \begin{bmatrix} y_0(\boldsymbol{\theta}) \\ y_1(\boldsymbol{\theta}) \\ \cdots \\ y_{63}(\boldsymbol{\theta}) \end{bmatrix}^{\mathrm{T}} (I^{\otimes 2} \otimes Z \otimes I^{\otimes 3}) \begin{bmatrix} y_0(\boldsymbol{\theta}) \\ y_1(\boldsymbol{\theta}) \\ \cdots \\ y_{63}(\boldsymbol{\theta}) \end{bmatrix} = z_0(\boldsymbol{\theta}), \tag{S2}$$

$$\langle \hat{Z}_5 \rangle = \langle Y|\hat{Z}_5|Y\rangle = \begin{bmatrix} y_0(\boldsymbol{\theta}) \\ y_1(\boldsymbol{\theta}) \\ \cdots \\ y_{63}(\boldsymbol{\theta}) \end{bmatrix}^{\mathrm{T}} (I^{\otimes 5} \otimes Z) \begin{bmatrix} y_0(\boldsymbol{\theta}) \\ y_1(\boldsymbol{\theta}) \\ \cdots \\ y_{63}(\boldsymbol{\theta}) \end{bmatrix} = z_1(\boldsymbol{\theta}). \tag{S3}$$

At this point, the actual output of the model can be represented as $\boldsymbol{y} = [z_0(\boldsymbol{\theta}), z_1(\boldsymbol{\theta})]$, where $z_0(\boldsymbol{\theta}), z_1(\boldsymbol{\theta}) \in [-1, 1]$. Submitting $\boldsymbol{y} = [z_0(\boldsymbol{\theta}), z_1(\boldsymbol{\theta})]$ to the *Softmax* function yields $\tilde{\boldsymbol{y}} = [\tilde{z}_0(\boldsymbol{\theta}), \tilde{z}_1(\boldsymbol{\theta})]$, where $\tilde{z}_0(\boldsymbol{\theta}), \tilde{z}_1(\boldsymbol{\theta}) \in [0, 1]$, and $\tilde{z}_0(\boldsymbol{\theta}) + \tilde{z}_1(\boldsymbol{\theta}) = 1$. Here, $\tilde{z}_0(\boldsymbol{\theta})$ and $\tilde{z}_1(\boldsymbol{\theta})$ represent the probabilities of the two classes.

Let the expected output be $\hat{\boldsymbol{y}} = [\hat{y}_0, \hat{y}_1]$, $\hat{y}_0, \hat{y}_1 \in \{0, 1\}$. Following Eq. (23) in the main text, the loss function value of this training sample is $C(\boldsymbol{\theta}) = -\hat{y}_0 \log \tilde{z}_0(\boldsymbol{\theta}) - \hat{y}_1 \log \tilde{z}_1(\boldsymbol{\theta})$.

The negative gradients of each model parameter can be calculated according to the following equation based on the gradient descent method,

$$-\frac{\partial C(\boldsymbol{\theta})}{\partial \theta_i} = -\frac{\partial(-\hat{y}_0 \log \tilde{z}_0(\boldsymbol{\theta}) - \hat{y}_1 \log \tilde{z}_1(\boldsymbol{\theta}))}{\partial \theta_i} = \hat{y}_0 \frac{1}{\tilde{z}_0(\boldsymbol{\theta})} \frac{\partial \tilde{z}_0(\boldsymbol{\theta})}{\partial \theta_i} + \hat{y}_1 \frac{1}{\tilde{z}_1(\boldsymbol{\theta})} \frac{\partial \tilde{z}_1(\boldsymbol{\theta})}{\partial \theta_i}. \tag{S4}$$

At this point, each model parameter can be updated according to the following equation,

$$\theta_i = \theta_i - \beta \frac{\partial C(\boldsymbol{\theta})}{\partial \theta_i}, i = 1, 2, \cdots, 10l + 6, \tag{S5}$$

where β is the learning rate.
